# Combining BET inhibition with SMAC mimetics restricts tumor growth and triggers immune surveillance in preclinical cancer models

**DOI:** 10.1016/j.xcrm.2025.102313

**Published:** 2025-08-25

**Authors:** Ksenija Slavic Obradovic, Florian Ebner, Artem V. Artemov, Martina Miotto, Paula-Elena Traexler, Robin Jacob, Ha Pham Thi Thanh, Regina Ruzicka, Andreas Wernitznig, Ines Baumann, Daniel Gerlach, Maria-Antonietta Impagnatiello, Salvatore Siena, Mary Murphy, Reniqua House, Ulrich Reiser, Valeria Santoro, Johannes Popow, Sebastian Carotta, Anke Baum, Jesse Lipp, Alberto Bardelli, Ulrike Tontsch-Grunt, Mariangela Russo, Martin Aichinger

**Affiliations:** 1 Boehringer Ingelheim RCV GmbH & Co KG, Vienna, Austria; 2 Department of Oncology, Molecular Biotechnology Center, University of Torino, Torino, Italy; 3 Boehringer Ingelheim Pharmaceuticals, Inc., Ridgefield, CT, USA; 4 IFOM ETS, The AIRC Institute of Molecular Oncology, Milano, Italy; 5 Department of Oncology and Hemato-Oncology, Università degli Studi di Milano, Milan, Italy; 6 Department of Hematology, Oncology, and Molecular Medicine, Grande Ospedale Metropolitano Niguarda, Milan, Italy

**Keywords:** SMAC mimetic, cIAP, BET inhibition, cell death, tumor microenvironment, combination therapy

## Abstract

Second mitochondrial activator of caspase (SMAC) mimetics (SMACm) and bromodomain and extra-terminal domain (BET) inhibitors (BETi) are two distinct classes of novel anticancer therapeutics. So far, broad clinical benefit for either monotherapy has not been achieved, calling for effective combination strategies.

We show that the combination of BI 891065, a monovalent oral SMACm antagonist of inhibitor of apoptosis protein 1 (cellular inhibitor of apoptosis protein 1 [cIAP1]), and BI 894999, a potent and selective oral BETi, significantly impaired cancer cell proliferation irrespective of tissue context.

Interestingly, we observed various forms of cell death pointing at distinct, but functionally converging, modulation of cell death-promoting pathways. A multi-omic analysis using Cellular Indexing of Transcriptomes and Epitopes by sequencing (CITE-seq) and advanced flow cytometry of a syngeneic model of pancreatic ductal adenocarcinoma (PDAC) unveils distinct phenotypic correlations of augmented anti-tumor immunity and a substantially reduced immunosuppressive tumor microenvironment (TME). Collectively, this study presents BETi and SMACm as a promising drug combination for patients with cancer with a multi-layered impact on both tumor cell-intrinsic and TME-dependent mechanisms.

## Highlights

• BETi+SMACm inhibits cancer cell proliferation resulting in enhanced *in vivo* efficacy
• Combination therapy modulates the tumor microenvironment and enhances anti-tumor immunity
• CITE-seq in syngeneic PDAC model provides insight into impact on anti-tumor immunity

## Graphical abstract

**Figure:**
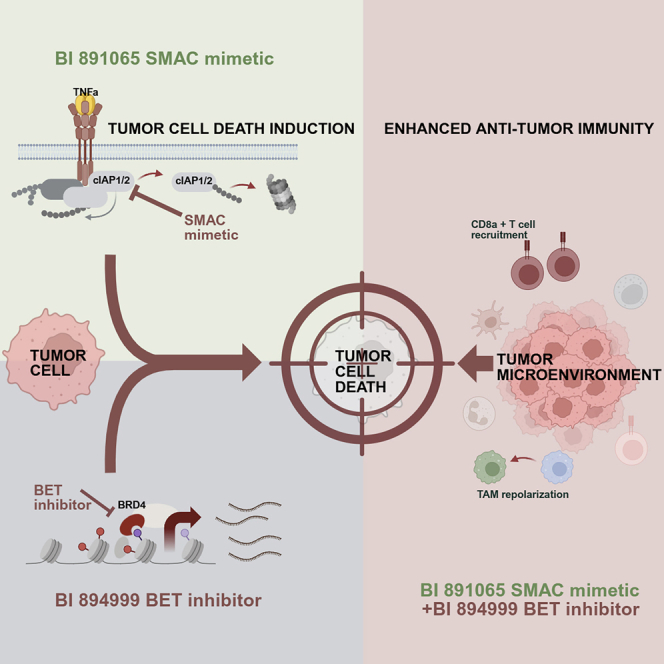

## Teaser

Slavic-Obradovic et al. demonstrate broad synergy between SMAC mimetics and BET bromodomain inhibition in inducing tumor cell death across solid cancer indications. In a pancreatic cancer model, they reveal that this combination remodels the tumor microenvironment and promotes anti-tumor immunity.

## Introduction

The discovery of effective combination therapies for cancer holds promise to broadening response rates to a wider indication spectrum as well as suppressing the emergence of drug resistance.^1^ In this study, we investigated the therapeutic potential of simultaneously targeting two protein families critical for proliferation and survival of cancer cells—the bromodomain and extra-terminal domain (BET) protein family of transcriptional regulators and inhibitors of apoptosis proteins (IAPs).

The BET protein family consists of BRD2, BRD3, BRD4, and BRDT. The best-studied BET family member BRD4 is a “reader” of epigenetic information and can bind to acetylated chromatin and non-chromatin proteins to act as a key regulator of transcription.^2^ Transcriptional dysregulation arising through altered activity of BET proteins leads to expression of multiple genes involved in carcinogenesis. Hence, inhibitors of BET proteins (BETi) have been tested in numerous clinical trials as a novel treatment opportunity for hematological malignancies and solid cancers.^3^^,^^4^^,^^5^ Mechanistically, BET small-molecule inhibitors displace BET bromodomains from acetylated proteins by competitively binding to the acetylated lysine recognition pocket. BI 894999 is an oral small-molecule BETi, previously tested in a phase 1 trial.^6^^,^^7^^,^^8^ However, as observed for numerous clinically investigated BETi, on-target adverse events (such as thrombocytopenia) and the emergence of resistance mechanisms limit the therapeutic potential of BETi as monotherapy.^8^^,^^9^ Thus, continued efforts are required to identify combinations, which would maximize the efficacy of BETi in cancer treatment.^10^^,^^11^

Small-molecule compounds mimicking the second mitochondrial activator of caspases (SMAC), SMAC mimetics (SMACm), are antagonists of IAPs and have been clinically evaluated as anti-cancer treatments.^12^ IAPs are often expressed at high levels in cancer, conferring resistance to apoptosis or other modalities of cell death.^13^ Among the best-characterized IAPs are cellular IAP1 (cIAP1 or BIRC2), cellular IAP2 (cIAP2 or BIRC3), and X chromosome-linked IAP (XIAP or BIRC4).^13^ All IAP members contain baculovirus IAP repeat (BIR) motifs promoting protein-protein interactions important for their anti-apoptotic activity, while cIAP1, cIAP2, and XIAP also contain a really interesting new gene domain conferring ubiquitin ligase (E3) activity.^14^ SMAC mimetics may antagonize the activity of IAPs by releasing XIAP from its inhibitory function against caspase-3, -7, and -9 and/or by stimulating the E3 ubiquitin ligase activity of cIAP proteins and thereby inducing auto-ubiquitination and degradation of cIAP1 and cIAP2 via the proteasome.^15^ By triggering the proteasomal degradation of cIAP proteins, SMACm consequently modulate ubiquitin-dependent signaling within the canonical nuclear factor κB (NF-κB) pathway upon tumor necrosis factor (TNF) signaling.^16^^,^^17^ Specifically, loss of cIAP 1 and 2 leads to deubiquitylation of receptor-interacting protein kinase 1 (RIPK1) and formation of a RIPK1/Fas-associated protein with death domain (FADD)/caspase-8 complex, activating caspase-8 and apoptotic cell death. Alternatively, RIPK1 can form a complex with RIPK3 in conditions when caspase-8 activity is compromised, leading to phosphorylation of mixed lineage kinase domain-like pseudokinase and induction of necroptosis.^18^^,^^19^^,^^20^ In addition to modulating canonical NF-κB signaling, degradation of cIAPs can lead to activation of the non-canonical NF-κB pathway and autocrine TNF signaling, resulting in induction of cell death.^16^^,^^17^

Mechanistically, both BETi and SMACm exert multifaceted effects on tumor cells, and their activity may converge at multiple points to initiate a cell death cascade. BETi have been shown to modulate the NF-κB pathway.^21^^,^^22^ Specifically, BRD4 was shown to maintain the active form of NF-κB in tumors,^23^ while BET inhibition broadly sensitized diverse tumor cell lines to the cytotoxic activity of TNF, involving the suppression of pro-survival NF-κB transcription.^23^^,^^24^^,^^25^ Previous reports of BETi-dependent downregulation of the anti-apoptotic genes CFLAR (encoding c-FLIP) and XIAP^26^ provide further rationale for combination treatments with SMACm. Indeed, downregulation of c-FLIP enhanced death of tumor cells upon SMACm treatment.^27^ Furthermore, BET inhibition has also been reported to suppress B cell lymphoma 2 (BCL-2),^28^ while BCL-2 downregulation or inhibition sensitized tumor cells to SMACm-induced apoptotic cell death.^23^^,^^24^^,^^25^^,^^28^^,^^29^

In addition to cancer cell-intrinsic activity, both BETi and SMACm have shown wide-ranging immunomodulatory effects, which may favor anti-tumor immune activity.^30^^,^^31^^,^^32^ In addition to the effects on the immune compartment, BET inhibition also induced stromal remodeling in pancreatic cancer, e.g., through suppression of tumor-promoting cancer-associated fibroblasts (CAFs).^33^ Here, we examined the effect of combining BI 894999 with BI 891065 *in vitro* across a large panel of human tumor cell lines (lung cancer, colorectal cancer [CRC], pancreatic cancer, and gastric cancer) and further investigated its consequences on cell death induction. We evaluated combination effects on tumor growth *in vivo* in five xenograft models. Finally, we explored the drug effects in the presence of a functional immune system, by employing a syngeneic model of pancreatic ductal adenocarcinoma (PDAC), Pan02. Using advanced single-cell multi-omic methods, we explored the drug impact on the tumor microenvironment (TME) and anti-tumor immunity and identified tumor cell-extrinsic phenotypic changes associated with treatment response.

## Results

### SMACm and BETi synergize in inducing a potent antiproliferative effect across a broad panel of cancer cell lines

BI 891065, a 2-amino-*N*-(6-ethynylpyridin-2-yl)propanamide, is a monovalent, oral SMACm with a favorable safety profile allowing continuous dosing and was previously evaluated in clinical phase 1 (NCT03166631 and NCT04138823).

**Figure 1:**
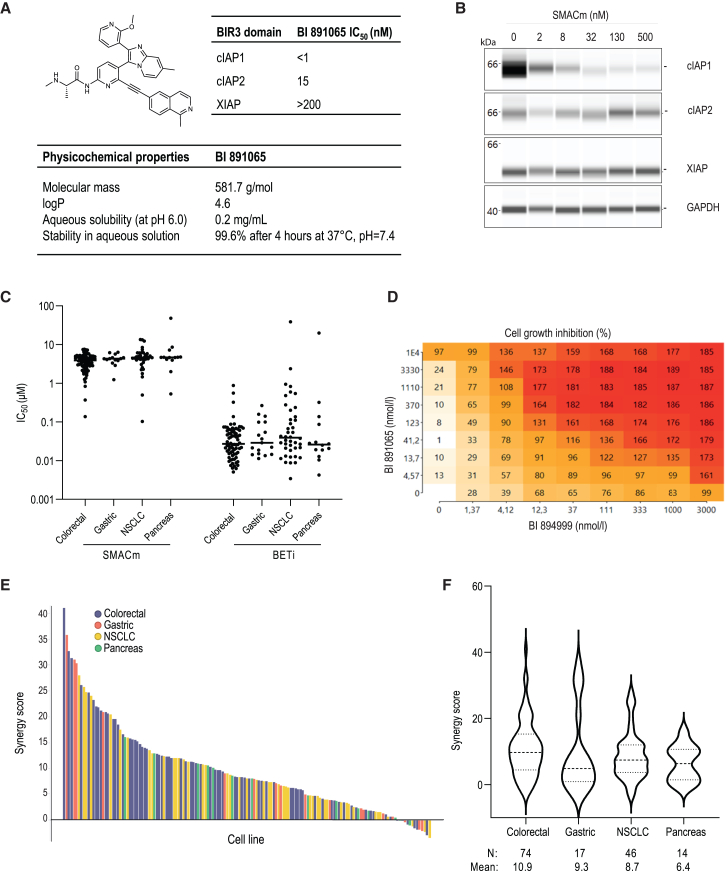
The combinatorial effect of BI 894999 and BI 891065 across a panel of human tumor cell lines (A) Selectivity and potency profiles of BI 891065. Top left, structure of BI 891065. Top right, IC_50_ values for BI 891065 inhibition of cIAP1, cIAP2, and XIAP. Bottom, physicochemical properties of BI 891965. (B) WES (Simple Western System) analysis of cIAP1, cIAP2, and XIAP levels upon treatment of HT-29 cells with indicated concentrations of BI 891065 for 6 h (C) IC_50_ values determined in cell proliferation assays (*n* = 151) individually for BETi BI 894999 and SMACm BI 891065. Each point represents a cell line tested. (D) Example of proliferation assay for synergy analysis in LoVo cell line; shown is combination plate map matrix and the cell growth inhibition (CGI) at the indicated concentrations; CGI values < 100%: tumor cell growth, CGI 100%: tumor cell stasis, and CGI > 100%: tumor cell killing. (E) Synergy scores across the tested panel of cell lines obtained using Bliss synergy analysis. (F) Violin plots showing the synergy scores for BETi and SMACm combination across indications. N is the number of cell lines tested in each indication group.

To determine the selectivity profile of BI 891065, binding assays were performed quantifying the interaction between BI 891065 and the baculoviral IAP repeat domain 3 domain of cIAP1, cIAP2, and XIAP by displacing the SMAC-derived peptide, as recently described for BI 878382.^32^^,^^34^ Overall, BI 891065 is chemically and functionally similar to BI 878382, exhibiting higher affinity toward cIAP1 compared to cIAP2 and more than 200-fold lower affinity toward XIAP (Figures 1A and 1B).

BI 894999 is a potent BET family inhibitor, blocking the binding of BRD2/3/4 and BRDT to acetylated histones in the low nanomolar range.^7^ BI 894999 has higher affinity toward bromodomain BRD4-BD1 compared to BRD4-BD2 (IC_50_ of 5 ± 3 nM and 41 ± 30 nM, respectively) and is 40- to 60-fold more potent than other BETi like JQ1 and molibresib (GSK525762).^8^

To investigate the synergistic interaction between BI 894999 (hereafter, BETi) and BI 891065 (hereafter, SMACm), we evaluated the *in vitro* anti-tumor effect of the single agents or the combination in a large panel of 151 cancer cell lines of different tissue origin: CRC, gastric cancer, non-small cell lung cancer (NSCLC), and pancreatic carcinoma (Figures 1C–1F). As a mono-agent, SMACm was able to inhibit cell proliferation only in a limited number of tested cell lines (4%, Table S2). Most of the cell lines responded to inhibition by BETi, with sensitivity ranging from 10 to 100 nM (Figure 1C). Bliss Gap analysis, performed to analyze the effect of the BETi+SMACm combination on tumor cell growth (Figures 1D–1F), unveiled potent synergistic activity across the cell models tested (Figure 1E), independent of tumor type (Figure 1F).

### SMACm and BETi combination can induce different modalities of cell death in cancer cells

**Figure 2:**
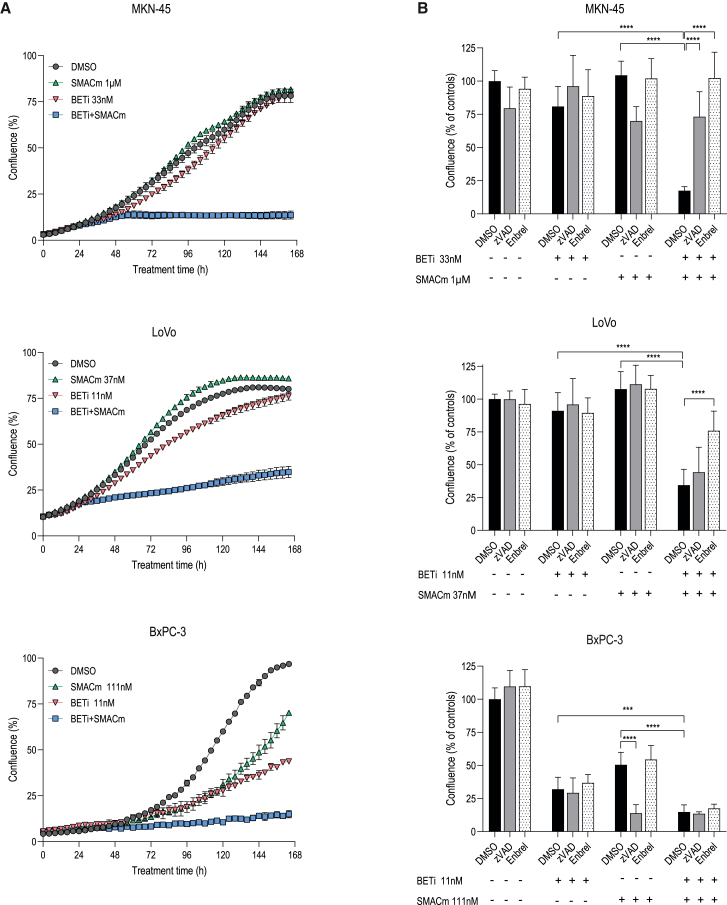
BETi and SMACm combination induces multiple forms of cell death *in vitro* (A) IncuCyte S3 live-cell imaging, following *in vitro* cell growth over time of three indicated human cell lines. Treatments were as shown and chosen based on the synergy analysis. Error bars indicate SEM of technical triplicates. Representative examples of 3 independently repeated experiments are shown. (B) Effect of TNF and caspase inhibition on induction of cell death by BETi and SMACm co-treatment. Shown is cell confluence measured at day 7 of treatment. Shown results are pool of 3 independent experiments (including data shown in A), plotted as mean +/- SD. BETi (BI 894999) and SMACm (BI 891065) concentrations were as shown in (A), TNF inhibitor Enbrel, 5 μg/mL, caspase inhibitor Z-VAD-FMK (zVAD), 20 μM. Statistical analysis: ∗∗∗*p* < 0.001, ∗∗∗∗*p* < 0.0001, one-way ANOVA with Šídák multiple comparison test.

The interaction between BETi and SMACm was further investigated mechanistically in three human cell lines. *In vitro* cell growth, assessed through live-cell imaging of BxPC-3 (human PDAC model), MKN-45 (human gastric adenocarcinoma model), and LoVo (human CRC model), showed a potent combinatorial effect across models tested leading to significant tumor cell growth inhibition or tumor cell death (Figure 2A).

To better characterize the mode of cell death induced by the combinatorial treatment, we evaluated the impact of caspase inhibition in this context. As both BETi and SMACm can lead to the production of TNF via an activation of the NF-κB pathway,^16^^,^^17^ we further substantiated our analysis by examining the role of TNF using the pharmacological decoy receptor Enbrel (etanercept).

MKN-45 cells under combination treatment were rescued from cell death by addition of a pan-caspase inhibitor (zVAD), as well as by blocking TNF signaling (Figure 2B), which indicates that MKN-45 cells undergo TNF-induced apoptosis. Consistent with this, we detected the cleavage of caspase-3 and poly (ADP-Ribose) polymerase (PARP) in lysates obtained from co-treated MKN-45 cells (Figure S1A). LoVo cells were rescued from cell death when TNF was blocked in the context of the BETi and SMACm combination treatment, while cell death induced in BxPC-3 cells upon combination treatment was independent of TNF (Figure 2B). zVAD had no effect on cell death induction in the LoVo and BxPC-3 cell lines, suggesting a modality of cell death different from that induced in MKN-45.

As XIAP, which is not effectively targeted by BI 891065 (Figure 1A), is one of the factors influencing the induction of cell death in the context of SMACm compounds,^13^ we monitored XIAP protein expression in the presence of mono- and combination treatment across a panel of 17 cell lines. Strikingly, XIAP was down-modulated by BETi in cell lines with synergistic activity of BETi and SMACm but not in those cell lines where the combination was not beneficial over monotherapy (Figures S1B and S1C).

Thus, our results show that depending on cellular context, combination of BETi and SMACm can lead to induction of various modalities of cell death that can differentially rely on TNF signaling.

### Combining SMACm and BETi is effective in colorectal patient-derived organoid models and in pancreatic and colorectal *in vivo* xenografts

**Figure 3:**
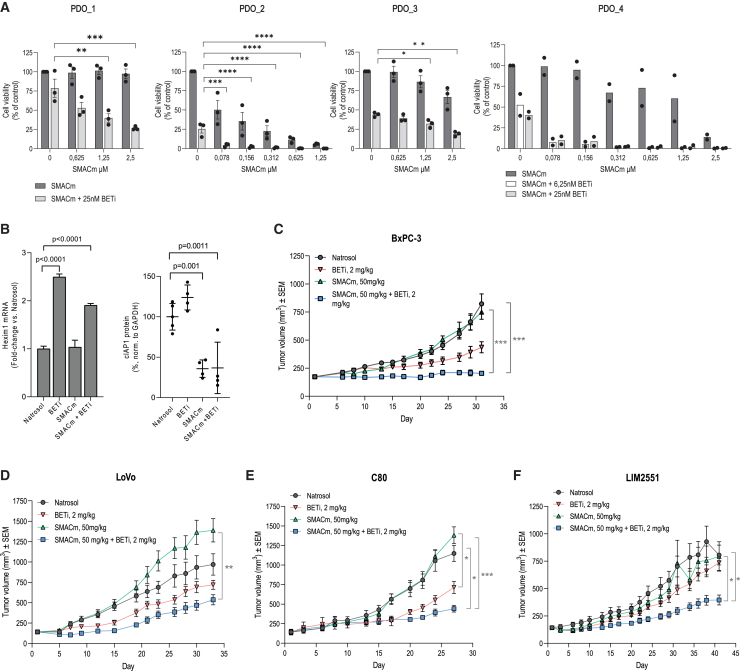
Efficacy of BETi and SMACm co-administration in CRC patient-derived organoid models and in PDAC and CRC cell line-derived xenografts (A) Sensitivity of CRC patient-derived organoids (PDOs) to increasing SMACm concentrations in the absence or presence of BETi. Average of three independent experiments for PDO_1, PDO_2, and PDO_3 and average of two independent experiments for PDO_4 are shown. For each experiment, data were normalized to the mean of the DMSO control wells and plotted as mean ± SEM values. (B) BETi and SMACm target engagement in BxPC-3 *in vivo* model, 4 h post treatment: Hexim1 mRNA upregulation upon BETi treatment; right, reduction in cIAP1 protein levels upon SMACm treatment. Plotted as mean +/- SEM values. (C–F) Mean tumor volumes +/- SEM in treated mice measured over a time course for different models: (C) pancreatic ductal adenocarcinoma BxPC-3 xenograft model (*p* < 0.001 for BETi+SMACm compared Natrosol and *p* < 0.001 for BETi+SMACm vs. SMACm monotherapy at the final time point, day 31). (D) LoVo colorectal carcinoma xenograft model (*p* = 0.003 BETi+SMACm compared to SMACm at the final time point d33). (E) C80 colorectal carcinoma xenograft model (*p* = 0.01 BETi+SMACm compared to Natrosol at the final time point, day 27; *p* < 0.001 BETi+SMACm compared to SMACm; *p* = 0.03 BETi+SMACm compared to BETi). (F) LIM2551 colorectal carcinoma xenograft model (*p* = 0.02 BETi+SMACm compared to Natrosol at the final time point day 41, *p* = 0.01 BETi+SMACm compared to SMACm). Statistics: (A and B) one-way ANOVA with Dunnett’s multiple comparison test (shown only for experiments with 3 independent biological replicates); (C–F) Kruskal-Wallis test with Dunn’s correction for multiple comparisons was performed on the last time points where at least 70% of animals remained in all the treatment groups. In all four models, treatments were given orally at indicated doses, with q.d. schedule. BETi, BI 894999; SMACm, BI 891065.

To further approximate the effect of the combinatorial regimen on human disease, we performed experiments in CRC patient-derived organoids (PDOs), which more closely recapitulate disease. To this aim, we tested six CRC PDOs *in vitro* with either mono- or combination therapy. Notably, we observed that while BETi impaired cell growth, the combinatorial approach showed stronger inhibitory effects on cell proliferation in four out of six tested PDO models (Figures 3A and S1D), thus corroborating our findings from the panel of tested cell lines.

To further extend upon the physiological relevance of the BETi and SMACm combination, we next evaluated the impact of mono- and combination therapies *in vivo* in five different mouse models for CRC and PDAC. Importantly, the combination effect was evaluated at clinically meaningful doses for both compounds. In order to evaluate *in vivo* target engagement, we employed the BxPC-3 xenograft model and measured pharmacodynamic biomarker modulation for both compounds. We found that HEXIM1, a previously established biomarker for BETi target engagement in tumors,^6^ was upregulated upon BETi monotreatment and BETi+SMACm co-treatment (Figures 3B and S3A), consistent with appropriate target engagement *in vivo.* This also demonstrates that target engagement *in vivo* was not compromised in combination treatment when compared to BETi monotherapy. Furthermore, combination treatment induced substantial changes to the transcriptomic landscape of BxPC-3 tumors beyond the expected global changes induced by BETi, with a striking upregulation of pathways involved in TNF alpha signaling, apoptosis, and interferon alpha response, among others (Figure S3). Additionally, cIAP1 protein levels were found to be reduced in tumors of mice treated with SMACm alone and BETi+SMACm, in line with the mode of action of BI 891065 (Figure 3B). Importantly, even though SMACm showed strong target engagement, this did not affect tumor growth in the monotherapy setting, whereas the combined activity of BETi and SMACm translated into significantly enhanced tumor growth inhibition (TGI) and prolonged tumor stabilization (Figure 3C).

In three CRC xenograft models tested (LoVo, C80, and LIM2551), neither of the inhibitors applied alone at clinically relevant doses resulted in pronounced TGI when compared to the vehicle control. However, all CRC models showed an improved response and significant TGI upon the combination treatment regimen (Figures 3D–3F).

Overall, models established in BomTac:NMRI-*Foxn1*^*nu*^ mice (BxPC-3 and LoVo) tolerated the daily oral administration of both compounds well, either as mono- or joint therapy (Figure S2). However, in NOD SCID models (C80 and LIM2551), combination treatment with BETi and SMACm led to a modest decline in bodyweight observed over the course of the study (Figure S2).

Together, our findings demonstrate the enhanced therapeutic impact of the BETi and SMACm combination in inducing stronger TGI over either monotherapy in different *in vivo* xenograft models of CRC and PDAC origin.

### SMACm and BETi combination impairs tumor growth in a pancreatic syngeneic model

Accumulating evidence points toward a broad spectrum of immunomodulatory effects mediated by BETi and SMACm as single agents.^30^^,^^31^^,^^32^^,^^33^^,^^35^^,^^36^ Encouraged by potent effects observed in human PDAC BxPC-3, we employed the syngeneic murine model of PDAC Pan02 to probe the impact of treatment on the immune compartment and the TME *in vivo*.

**Figure 4:**
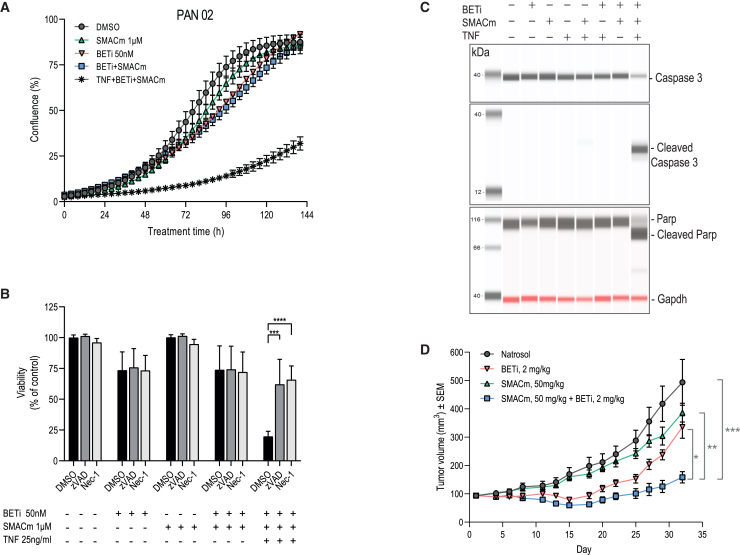
BETi and SMACm combination impairs tumor growth of a pancreatic syngeneic model (A) Time course of *in vitro* cell growth for mouse cell line Pan02 measured by IncuCyte S3 live-cell imaging. Treatments were as indicated. Mouse TNF concentration was 25 ng/mL. Representative example of 3 repeated experiments is shown. Error bars indicate SEM of technical triplicates. (B) Caspase inhibition by Z-VAD-FMK (zVAD) and RIPK1 inhibition by necrostatin-1 (Nec-1) rescue Pan02 cells from cell death induced by BETi+SMACm combination in the presence of TNF. Cell viability was measured by the CellTiter-Glo assay 96 h after treatments started. Shown results are pool of 2 independent experiments. Concentration of zVAD was 20 μM, and Nec-1 concentration was 10 μM (one-way ANOVA with Dunnett’s multiple comparison test; ∗∗*p* < 0.01). (C) JESS (Simple Western System) analysis of caspase-3 and PARP cleavage in Pan02 cell line upon treatments with BETi (50 nM), SMACm (1 μM), and TNF (25 ng/mL). Cell pellets were collected for lysis and JESS analysis at the 24 h time point after treatments started. Error bars indicate SEM. (D) Mean tumor volumes of Pan02 syngeneic model (*p* < 0.001 for BETi+SMACm compared to Natrosol at the final time point, day 32; *p* = 0.002 for BETi+SMACm vs. SMACm monotreatment; and *p* = 0.04 for BETi+SMACm vs. BETi monotreatment). Statistics: Kruskal-Wallis test with Dunn’s correction for multiple comparisons was performed on the last time points where at least 70% of animals remained in all the treatment groups. Treatments were given orally at indicated doses, with q.d. schedule. BETi, BI 894999; SMACm, BI 891065.

Initial *in vitro* characterization of Pan02 unveiled synergy for BETi and SMACm combinatorial activity only in the context of exogenously supplied TNF (Figure 4A). We found Pan02 cells to be resistant to even high concentrations of TNF alone, TNF plus SMACm, or TNF plus BETi (Figure S1E). However, in the presence of even low TNF concentrations, a strong synergistic effect was observed for BETi and SMACm combinatorial administration (Figures S1E and S1F).

This led us to further investigate the mechanism of cell death induced in Pan02 cells. Our findings point to RIPK1-dependent apoptosis, as cells were rescued from cell death by addition of the caspase inhibitor zVAD or the RIPK1 inhibitor Nec-1 (Figure 4B). In agreement with the induction of apoptotic cell death, we detected the cleavage of caspase-3 and PARP in lysates obtained from cells treated with the combination in the presence of TNF (Figure 4C).

*In vivo* assessment further validated the potential of this combination showing a strong effect when treating with SMACm + BETi in this immunocompetent model. The efficacy observed *in vivo*, together with the crucial dependence on exogenous TNF *in vitro*, suggests an essential contribution of cues provided by cells present within the TME to the efficacy of combination treatment (Figure 4D).

### Combining SMACm and BETi induces immune-modulatory effects in an immunocompetent pancreatic (Pan02) model

In order to dissect the effects of SMACm and BETi therapy on the immune compartment and the TME, we performed an additional *in vivo* experiment in the syngeneic Pan02 (Figures S4B, 5A, and 5B) model using flow cytometry and Cellular Indexing of Transcriptomes and Epitopes by Sequencing (CITE-seq) as readouts (Figure S4A).

**Figure 5:**
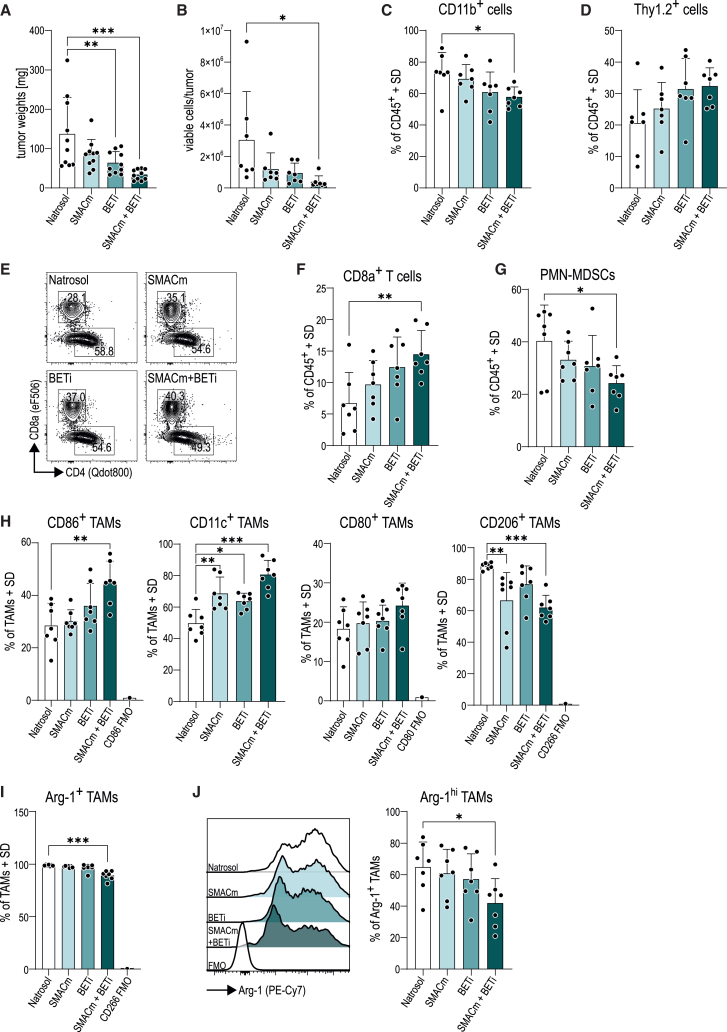
Combined SMACm and BETi treatment augments anti-tumor immunity *in vivo* (A) Tumor weights on the day of analysis (day 16 post treatment, *n* = 10/group). (B) Viable cell numbers determined by trypan blue-stained automated counting. (C and D) Fraction of (C) myeloid cells (CD11b+) and (D) T cells (Thy1.2+) as a percentage of all immune cells pre-gated on living CD45^+^. (E) Representative flow cytometry plots show CD8a+ and CD4^+^ cell frequency within the Thy1.2+ population. (F) CD8a+ T cell frequency among all immune cells. CD8a+ T cells were pre-gated as living CD45+Thy1.2+. (G) Frequency of polymorphonuclear myeloid-derived suppressor cells (PMN-MDSCs) among all immune cells gated on living CD45^+^CD11b+Siglec-F-Ly6CmedLy6G+. (H) Macrophage polarization marker expressing cells as frequency of all tumor-associated macrophages (TAMs). Depicted are CD86, CD11c, CD80, and CD206 (from left to right). (I) Frequency of arginase-1-expressing TAMs among all TAMs. TAMs were gated on living CD45^+^CD11b+Siglec-F-Ly6C-F4/80+. (J) Left, histograms showing arginase-1 high- and low-expressing populations of macrophages. Right, bar plots show Arg-1hi-expressing TAMs among all Arg-1+ TAMs. *n* = 7/group. Data plotted in A–D and F–J are ±/− SD. Statistical significance: ∗*p* < 0.05; ∗∗*p* < 0.01; ∗∗∗*p* < 0.001; no label, not significant.

Multicolor spectral flow cytometry was performed with a focus on analysis of tumor-infiltrating immune cells (Figure S4; Table S4). Our data show that CD11b+ myeloid cells were significantly reduced upon combination treatment (Figure 5C), and we observed a trend toward increased frequency of T cells (Figure 5D). Within the T cell compartment, the fraction of cytotoxic CD8a+ T cells was significantly increased in the combination arm (Figures 5E and 5F), while frequencies of helper T cells (CD4^+^CD25−Foxp3−) and regulatory T cells (CD4^+^CD25+Foxp3+) remained unaffected (Figure S4J).

Within the myeloid cell population, the combined action of SMACm and BETi leads to a substantial reduction of immunosuppressive polymorphonuclear myeloid-derived suppressor cells (PMN-MDSCs)/neutrophils (Figure 5G).

Frequencies of eosinophils and tumor-associated macrophages (TAMs) were largely comparable across all treatment groups, whereas monocytic MDSCs and myeloid dendritic cells were more abundant in the double-treated animals (Figure S4I). Although TAM frequency was largely similar across groups, we observed differences in markers for their polarization. Frequencies of pro-inflammatory TAMs expressing CD86 and CD11c were increased upon double treatment (Figure 5H). In contrast, the frequency of TAMs expressing the anti-inflammatory marker CD206 was reduced upon SMACm mono- and SMACm + BETi treatment (Figure 5H).

Additionally, we investigated the expression of the immunosuppressive enzyme arginase-1^37^ and found a reduction of Arg-1+ TAMs within tumors from the double-treated cohort (Figures 5I and 5J).

Endothelial cell and cancer-associated fibroblast (CAF) frequencies within the TME were similar across all treatment groups (Figure S4G). However, the CAF sub-population expressing the marker Ly-6C, previously associated with a tumor-promoting inflammatory CAF^38^ phenotype, was strongly reduced in the co-treated tumors (Figure S4H).

Taken together, this flow cytometry analysis demonstrated distinct pharmacodynamic changes elicited by BETi+SMACm co-treatment which correlated with increased tumor control, enhanced CD8a+ T cell immunity, and reduced immunosuppressive cell types—such as PMN-MDSCs, arginase-1-expressing TAMs, and Ly-6C+ CAFs.

### Single-cell CITE-seq analysis showed improved anti-tumor immunity and a reduced immunosuppressive TME in the syngeneic Pan02 preclinical model

To further examine therapy effects on the immune compartment of Pan02 tumors with a single-cell resolution, we next performed multi-omic CITE-seq analysis aiming at deeper characterization of immune cell correlates of efficacy in response to BETi+SMACm treatment.

**Figure 6:**
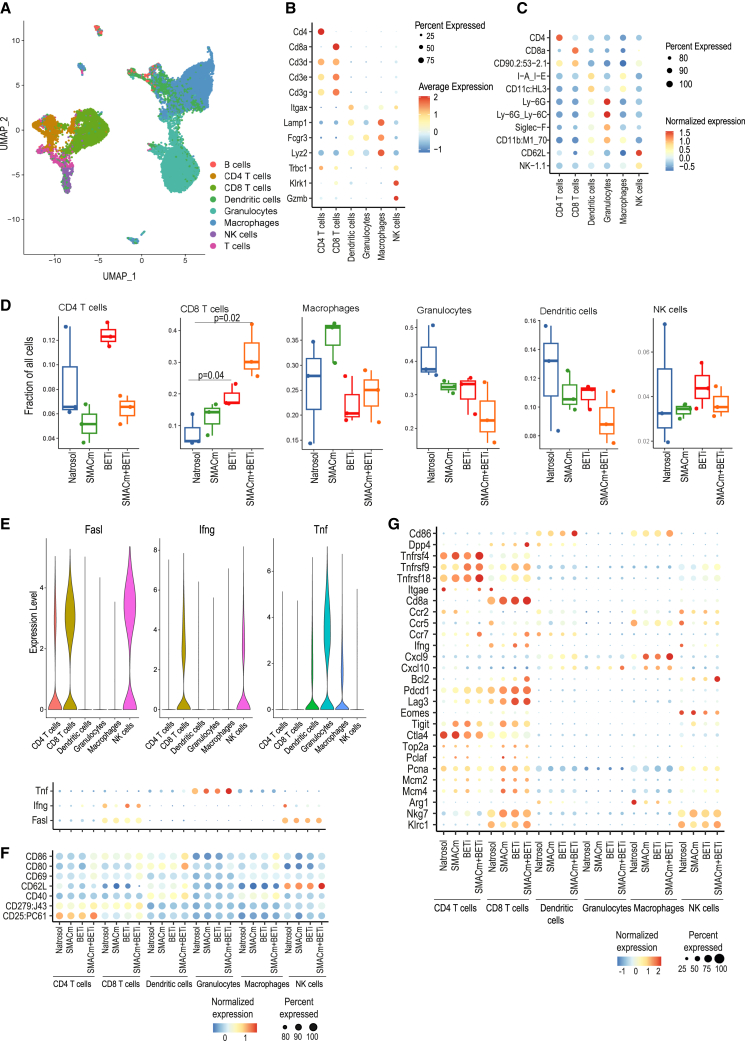
Immune-modulatory effects of SMACm and BETi in the syngeneic PDAC model (A) Uniform manifold approximation and projection cell clustering based on CITE-seq RNA and protein analysis. (B and C) (B) Gene expression (single-cell RNA sequencing) and (C) surface protein abundance (CITE-seq) of cell type markers. (D) Fractions of cell types shown in (A) split by treatments; fractions were calculated as a ratio between the number of cells in a cell type and the total number of cells in the treatment. (E) Violin plots showing expression of selected cytotoxic cytokines across cell types shown in (A) (top). Bottom, expression of Tnf, Ifng, and Fasl RNA across cell types split by treatments assessed by CITE-seq analysis (gene expression is normalized by Seurat in a standard way). Order of samples is corresponding to the dot plot below in (F). (F) Dot plot shows selected top differentially abundant surface proteins assessed by CITE-seq (average row-wise expression was subtracted for normalization). (G) Gene expression profiling by CITE-seq analysis; heatmap shows RNA expression of selected top differentially expressed genes (DEGs) across main immune cell types split by treatment.

To this end, we quantified 56 cell surface proteins and mRNA expression levels of 397 immune response genes. Dimensionality reduction of obtained multi-omic data allowed identification of cell states and the annotation of landmark immune cell subsets (Figures 6A–6C). Treatment impact on tumor-infiltrating leukocyte composition was in agreement with the flow cytometry analysis (Figure 5). In co-treated tumors, CD8^+^ T cell frequency increased, while a trend toward decreased frequency of dendritic cells and granulocytes was observed (Figure 6D).

As the presence of TNF is necessary for the antiproliferative effect of BETi+SMACm on Pan02 cells *in vitro* (Figure 4A), we scouted for the cellular sources of *Tnf* mRNA expression within the TME. Among immune cells, granulocytes were found to be the most prominent producers of *Tnf* mRNA, and importantly, *Tnf* expression levels were highest upon BETi+SMACm co-treatment (Figure 6E). In contrast, the expression of Fas ligand on CD8^+^ T cells and natural killer (NK) cells was largely unaffected by drug treatment (Figure 6E). *Ifng* expression was highest in CD8^+^ T cells obtained from BETi-treated tumors and control NK cells (Figure 6E).

We further interrogated expression patterns of the most differentially expressed genes across main immune cell subsets (Figure 6G; Table S5). T cells from BETi+SMACm-treated tumors expressed higher levels of genes implicated in T cell activation: *Cd8a*, *Tnfrsf4* (*OX40*), *Tnfrsf9* (*4-1BB*), *Tnfrsf18*, *Dpp4*, and *Ccr7*^39^^,^^40^^,^^41^^,^^42^ (Figure 6G). CD8^+^ T cells further expressed genes involved in DNA replication (*Top2a*, *Pcna*, *Mcm2*, *Mcm4*, and *Bcl2*) indicating a proliferative state, together with increased expression of *Pdcd1* (encoding for PD-1) and *Lag3* as a consequence of T cell activation (Figure 6G).

In line with these indicators of increased activity, the immune checkpoint *Ctla4* was downregulated in CD8^+^ T cells upon double treatment. Expression of *Cd86*, a key co-stimulatory factor involved in T cell activation and survival, was strongly induced in dendritic cells upon combination treatment (Figure 6G).

Expression of chemokines and cytokines promoting the recruitment of effector T cells and natural killer cells into tumors,^43^^,^^44^ such as *Cxcl9* and *Cxcl10*, was prominently induced in macrophages obtained from the BETi+SMACm-treated tumors. In agreement with our flow cytometry analysis, the expression of *Arginase-1* mRNA was downregulated in macrophages isolated from co-treated tumors. In line with these observations, macrophage expression of both *Ccr2* and *Ccr5*, implicated in the recruitment of immunosuppressive myeloid cells into the TME,^45^^,^^46^ was also reduced (Figure 6G).

In addition to mRNA expression analysis, the CITE-seq approach facilitated the simultaneous examination of cell surface protein abundance (Figures 6F and S5). Consistent with polarization toward M1 phenotype, macrophages and dendritic cells exhibited upregulation of the pro-inflammatory cell surface markers CD86 and CD80 in the SMACm + BETi treatment group (Figures 6F, S5C, and S5E). Correspondingly, the augmented activation of CD8a+ T cells observed at the mRNA level was reflected in the highest surface protein expression of activation markers CD69, CD279 (PD-1), and CD25 (IL-2Ra) in the combination treatment group (Figures 6F and S5B). Interestingly, the previously noted down-regulation of PD-L1 expression^47^ in tumor cells following BET inhibition was not evident in immune cells within this experimental setup. On the contrary, PD-L1 (CD274) protein expression was consistently elevated across all immune cell types (Figures S5A–S5F).

Collectively, our CITE-seq analysis complements the cytometry findings described earlier by adding a functional layer building on gene and protein expression data at the single-cell level. The induction of TNF and genes implicated in T cell activation and proliferation together with a distinct modulation of immune-suppressive cues further corroborates the anti-tumor activity of BETi+SMACm combination exerted via the immune compartment.

## Discussion

Despite broad evidence for the anticancer efficacy of BETi in preclinical studies, efforts to translate these findings for therapeutic application have been hampered by side effects and a short duration of clinical response. These limitations call for the identification of effective combination drugs, which may allow lowering therapeutic doses.^9^ SMACm promote apoptosis in cancer cells by antagonizing the antiapoptotic effects of IAPs. Here, we report the structure of a novel small-molecule SMACm (BI 891065) and an in-depth exploration of the combination with a BETi (BI 894999) in diverse solid cancer models (lung, CRC, pancreatic, and gastric cancer). Additionally, synergistic activity between SMAC mimetic and BET inhibition has also been reported in the context of HIV infection,^48^ thereby underscoring the potential of this combination in cancer and beyond. The relevance of this novel combination is underlined by a broad and tissue type-independent response pattern within a cohort of 151 cell lines, thus suggesting that this combination may be applied across solid cancers. Of note, the translational implications of these findings were further confirmed by demonstrating the efficacy in clinically relevant organoid models derived from patients with CRC.^49^^,^^50^^,^^51^

Mechanistically, our data portray a unique property of this therapeutic approach to robustly facilitate different forms of cell death like apoptosis (MKN-45 and Pan02) or caspase-independent forms of cell death (LoVo and BxPC-3). Adding to this robustness is the compatibility of this therapeutic modality with several independent cell death triggers. As it is fair to assume that in our experimental *in vitro* screening setup not all physiological cell death triggers are available, we solely rely on factors produced by tumors cells in an autocrine or paracrine fashion. From human cell lines tested here, almost 30% revealed synergistic activity of BETi and SMACm, even without exogenous supplementation of TNF or other cell death ligands. Under physiologic conditions, sources of cell death inducing signals may be pleiotropic and may come from a multitude of different cell types present in the TME. Consequently, our extensive *in vitro* drug sensitivity assessment is likely to underestimate the full potential of combining BETi with SMACm.

Our experiments clearly demonstrate an essential role for TNF in being the initial cell death trigger for some cell lines (MKN-45, LoVo, and Pan02); cell death in other cell lines like BxPC-3 is initiated by factors other than TNF, as functional depletion did not impact the viability of cells in the context of BETi+SMACm treatment. Importantly, SMACm have been shown to facilitate death induction in the presence of other cell death ligands, e.g., interferons and TNF-related apoptosis inducing ligand (TRAIL),^52^ while BET-degrading compounds were previously shown to upregulate TRAIL receptor 2 (TRAILR2) and thereby induce cell death in CRC cell lines.^53^

TNF and other death ligands can—in principle—be provided by many cells within the TME, including autocrine secretion from cancer cells upon stimulation,^32^^,^^54^ cytotoxic T cells, but potentially also other cells within the TME. Importantly, immune cells can be key providers of such cell death triggers,^55^ and to experimentally evaluate the complex interplay and exchange of relevant signaling cues upon treatment, we employed the syngeneic cell line Pan02. In this model, we show that extrinsic cell death ligands can indeed be found in the TME and in the case of TNF are upregulated upon SMACm and BETi treatment.

Further leveraging state-of-the-art multi-omic analysis, we found that SMACm and BETi combination overall shaped the TME to be less immunosuppressive. This state is relayed via a concerted treatment-induced modulation of key cell types and phenotypes. While generally these effects were most pronounced in the combination setting, some effects were primarily driven by either BETi or SMACm treatment. For example, SMACm treatment was the driving cause of a reduction of M2-polarized TAMs, while at the same time leading to an increased abundance of TAMs with a pro-inflammatory signature (highlighted by increased expression of CD86 and CD11c protein, *Cxcl9* and *Cxcl10* mRNA, and reduced levels of Arg-1 protein and mRNA^56^^,^^57^^,^^58^). As Arg-1 expression of TAMs was previously shown to be a central driver of immunosuppression in pancreatic cancer by inhibiting CD8^+^ T cell recruitment,^59^ this modulation is in agreement with increased immune cell activity, most prominently the increased frequencies of cytotoxic CD8^+^ T cells, and superior efficacy in the BETi+SMACm cohort.

Other favorable changes potentially contributing to the mechanisms affording enhanced tumor control were the reduction of additional tumor-promoting immunosuppressive cell types like Ly-6C+ CAFs and PMN-MDSCs.^38^^,^^44^ Interestingly, neither our experiments in the Pan02 model (Figures 5D and S4) nor in the BxPC-3 model (Table S3) did recapitulate the previously reported BETi-mediated suppression of programmed cell death protein 1 (PD-1)/programmed death ligand 1 (PD-L1) signaling.^30^^,^^35^

Taking into account our findings *in vivo*, in both immunocompromised and immunocompetent mouse models, we hypothesize that, in a clinical setting, both the cancer cell-targeted and immune-mediated effects of BETi and SMACm indeed operate in concert to improve anti-tumor activity, which may be exploited as an effective treatment in solid cancer of diverse tissue origin.

Clinical implications, beyond indications investigated here, include head and neck squamous cell carcinoma (HNSCC) for which there is a high unmet medical need for effective targeted therapies. Recent studies have shown great promise for IAP antagonists in the treatment of HNSCC, especially in combination with radiation therapy.^60^ Moreover, BRD4 is highly expressed in HNSCC, and BET inhibition has shown to increase anti-tumor immunity in HNSCC by enhancing major histocompatibility complex class I expression.^61^ These, together with our findings, would warrant investigation of the BETi and SMACm combination in the treatment of HNSCC, and the identification of patient selection biomarkers for this combination could further advance this concept for clinical application.

Taken together, our data put forward the promising therapeutic concept of combining BETi and SMACm. The simultaneous induction of tumor cell death and the modulation of anti-tumor immunity may ultimately lead to improved outcomes for patients with cancer.

### Limitations of the study

This study has limitations. We found that the combination of SMACm and BETi is effective in a substantial fraction of tumors independently of their tissue origin and genetic background, but we have not benchmarked this combination against other potential preclinical combinations and have not identified biomarkers associated with sensitivity or resistance. Our study positions TNF signaling as a central factor facilitating the anti-tumor efficacy of the combination treatment. However, in the BxPC3 model, the dominant effect is mediated by other factors, which we do not further characterize in this study. In the syngeneic PAN02 model, we describe various cellular sources of TNF, and while the presented data strongly suggest causality, we do not formally show the consequences of *in vivo* depletion of TNF. The associated single-cell analysis of the TME performed with subcutaneous PAN02 tumors represents one approach to evaluate changes within the TME in the context of treatment. Due to the lack of fully standardized methods in the field, certain aspects and analysis strategies may deviate across studies; however, our complementary analysis using flow cytometry and CITE-seq do alleviate such concerns. Our study deeply portrays the complex biology of SMACm + BETi treatment at the interface between tumor cells and the immune system, suggestive of an added benefit when combining with immune-targeted agents. We have not extended our mechanistic work in this direction.

## Resource availability

### Lead contact

Requests for further information or reagents should be directed to the lead contact, Martin Aichinger (martin.aichinger@boehringer-ingelheim.com).

### Materials availability

There are restrictions to the availability of materials. The PDOs are available from A. Bardelli and M.R. (UNITO) (MTA required). BI 894999 is available through opnME https://www.opnme.com/molecules/bet-inhibitor-bi89499.

### Data and code availability

• Single-cell sequencing data generated during the study are available at GEO accession number GSE269602.
• The codes used for the analyses are available at https://github.com/Boehringer-Ingelheim/smacm_beti_cellreportsmedicine.
• Any additional information required to reanalyze the data reported in this work paper is available from the lead contact upon request.

## Acknowledgments

We would like to acknowledge A. Krolo, J. Klufa, S. Kupka, and I. Tirapu for continued discussions, S. Olt and R. Holly for technical assistance, and E. Strauss for help with screening data import and validation.

Research leading to these results was supported by AIRC under 5 per Mille 2018 - ID. 21091 program – P.I. A. Bardelli; AIRC under IG 2023 ID. 28922 project – P.I. A. Bardelli; and AIRC under MFAG 2021 - ID 26439 project – P.I. M.R. The graphical abstract and Figure S4A were generated with BioRender.

## Author contributions

K.S.O., F.E., P.-E.T., H.P.T.T., R.R., R.J., I.B., M. Miotto, and M. Murphy performed the experiments. Conceptualization, analysis, and interpretation of *in vitro* experiments, K.S.O., A.W., V.S., J.P., S.S., D.G., F.E., M. Miotto, M. Murphy, R.H., M.A., M.R., and U.T.-G.; conceptualization, analysis, and interpretation of *ex vivo* data, M.A., F.E., A.V.A., J.L., V.S., and R.H.; conceptualization, analysis, and interpretation of *in vivo* experiments, A. Baum, U.T.-G., M.A., F.E., J.L., M.R., and A. Bardelli; BI 891065 design and synthesis, U.R.; BI 891065 development and characterization, M.-A.I. S.C. and U.T.-G. reviewed and edited the manuscript and provided key intellectual input. K.S.O., F.E., A.V.A., A. Bardelli, M.R., and M.A. wrote the manuscript.

## Declaration of interests

A. Bardelli declares the following competing financial interests: receipt of grants/research supports from Neophore, AstraZeneca, and Boehringer Ingelheim; receipt of honoraria or consultation fees from Guardant Health; stock shareholder at Neophore and Kither Biotech; and a member of the SAB of Neophore.

S.S. is an advisory board member for Agenus, AstraZeneca, Bayer, Bristol Myers Squibb, CheckmAb, Daiichi-Sankyo, GlaxoSmithKline, Istituto Nazionale Genetica Medica, MSD, Merck, Novartis, Ospedale San Raffaele, Pierre-Fabre, Pfizer, Seagen, and T-One Therapeutics. K.S.O., F.E., A.V.A., P.-E.T., R.J., H.P.T.T., R.R., A.W., I.B., D.G., M.-A.I., M. Murphy, R.H., U.R., V.S., J.P., S.C., A. Baum, J.L., U.T.-G., and M.A. are current or past employees of Boehringer Ingelheim. BI 894999 is part of patent applications derived from WO2014/076237, and BI 891065 is part of patent applications derived from WO2016/023858.

## STAR★Methods

### Key resources table

**Table:** 

REAGENT or RESOURCE	SOURCE	IDENTIFIER
**Antibodies**		

Thy1.2	BD	CAT# 740205; RRID:AB_2739955
CD45	BD	CAT# 749889; RRID:AB_2874129
CD31	BD	CAT# 741505; RRID:AB_2870961
CD80	BD	CAT# 741956; RRID:AB_2871264
F4/80	Biolegend	CAT# 123131; RRID:AB_10901171
CD11b	BD	CAT# 566117; RRID:AB_2739519
CD8a	Thermo Fisher	CAT# 69-0081-82; RRID:AB_2637161
CD44	Biolegend	CAT# 103037; RRID:AB_10900641
CD25	BD	CAT# 563061; RRID:AB_2737982
Ly-6C	Biolegend	CAT# 128037; RRID:AB_2562630
CD86	BD	CAT# 747439; RRID:AB_2872120
CD4	Thermo Fisher	CAT# Q22165; RRID:AB_2556521
CD11c	Biolegend	CAT# 117364; RRID:AB_2832410
MHCII (I-A/I-E)	Biolegend	CAT# 107624; RRID:AB_2191073
PD-L1	Biolegend	CAT# 116130; RRID:AB_2800583
Ly-6G (self-conjugated)	Biolegend/Thermo Fisher	CAT# 127601/K06T04L007
CD103	BD	CAT# 562772; RRID:AB_2737784
Siglec-F	BD	CAT# 562680; RRID:AB_2687570
Foxp3	BD	CAT# 563902; RRID:AB_2630318
CD206	Biolegend	CAT# 141738; RRID:AB_2860694
Arg1	Thermo Fisher	CAT# 25-3697-82; RRID:AB_2734841
CD3	BD	CAT# 940107; RRID:AB_2875997
CD4	BD	CAT# 940108; RRID:AB_2875998
CD45R (B220)	BD	CAT# 940110; RRID:AB_2876000
CD19	BD	CAT# 940111; RRID:AB_2876001
CD11c	BD	CAT# 940112; RRID:AB_2876002
Ly-6G	BD	CAT# 940113; RRID:AB_2876003
CD25	BD	CAT# 940116; RRID:AB_2876006
Siglec-F	BD	CAT# 940117; RRID:AB_2876007
Ly-6G/Ly-6C	BD	CAT# 940119; RRID:AB_2876009
CD28	BD	CAT# 940120; RRID:AB_287601
NK-1.1	BD	CAT# 940121; RRID:AB_2876011
CD62L	BD	CAT# 940122; RRID:AB_2876012
I-A/I-E	BD	CAT# 940123; RRID:AB_2876013
TCR BETA CHAIN	BD	CAT# 940125; RRID:AB_2876014
CD69	BD	CAT# 940126; RRID:AB_2876015
CD117 (c-Kit)	BD	CAT# 940127; RRID:AB_2876016
CD279 PD-1	BD	CAT# 940128; RRID:AB_2876017
CD86 (B7-2)	BD	CAT# 940129; RRID:AB_2876018
TER-119	BD	CAT# 940132; RRID:AB_2876021
CD103	BD	CAT# 940136; RRID:AB_2876025
CD90.2	BD	CAT# 940139; RRID:AB_2876028
CD335(NKp46)	BD	CAT# 940140; RRID:AB_2876029
CD80 (B7-1)	BD	CAT# 940141; RRID:AB_2876030
CD274 (B7-H1)	BD	CAT# 940142; RRID:AB_2876031
CD40	BD	CAT# 940143; RRID:AB_2876032
CD24	BD	CAT# 940144; RRID:AB_2876033
CD223 (LAG-3)	BD	CAT# 940152; RRID:AB_2876041
H-2Kb	BD	CAT# 940164; RRID:AB_2876052
CD197 (CCR7)	BD	CAT# 940165; RRID:AB_2876053
CD326 (Ep-CAM)	BD	CAT# 940169; RRID:AB_2876056
CD1d	BD	CAT# 940171; RRID:AB_2876058
CD54 (ICAM-1)	BD	CAT# 940172; RRID:AB_2876059
CD14	BD	CAT# 940174; RRID:AB_2876061
CD278(ICOS)	BD	CAT# 940176; RRID:AB_2876063
CD273 (PD-L2)	BD	CAT# 940180; RRID:AB_2876066
H-2Kd	BD	CAT# 940194; RRID:AB_2876077
CD115	BD	CAT# 940198; RRID:AB_2876081
CD137 (4-1BB)	BD	CAT# 940199; RRID:AB_2876082
CD172a (SIRPα)	BD	CAT# 940201; RRID:AB_2876084
CD45RB	BD	CAT# 940206; RRID:AB_2876088
CXCR2	BD	CAT# 940209; RRID:AB_2876091
TCR Vγ1.1	BD	CAT# 940334; RRID:AB_2876207
CD45Ra	BD	CAT# 940335; RRID:AB_2876208
CD8a	BD	CAT# 940345; RRID:AB_2876217
CD25	BD	CAT# 940356; RRID:AB_2876227
CD83	BD	CAT# 940350; RRID:AB_2876221
CD272	BD	CAT# 940415; RRID:AB_2876275
Vγ 3 TCR	BD	CAT# 940354; RRID:AB_2876225
F4/80-L	BD	CAT# 940412; RRID:AB_2876273
CD44	BD	CAT# 940114; RRID:AB_2876004
CD11b	BD	CAT# 940008; RRID:AB_2875899
cIAP1	Cell Signaling	CAT# 7065; RRID:AB_10890862
cIAP2	Cell Signaling	CAT# 3130; RRID:AB_10693298
XIAP	Cell Signaling	CAT# 14334; RRID:AB_2784533
GAPDH	Abcam	CAT# 8245; RRID:AB_2107448
PARP	Cell Signaling	CAT# 9542; RRID:AB_2160739
Caspase-3	Cell Signaling	CAT# 14220; RRID:AB_2798429
Cleaved Caspase-3	Cell Signaling	CAT# 9664; RRID:AB_2070042
Goat anti-Mouse IgG (H + L) Secondary Antibody DyLight 650	Novus	NBP1-75147C; RRID:AB_3219020

**Chemicals, peptides, and recombinant proteins**		

Z-VAD-FMK	Sigma-Aldrich	V116-2MG
Etanercept (Enbrel ®)	Pfizer	
Necrostatin-1	MedChemExpress	HY-15760
BI 894999	Boehringer Ingelheim	
BI 891065	Boehringer Ingelheim	
Halt™ protease and phosphatase inhibitor cocktail	Thermo Fisher Scientific	78440
MSD Tris Lysis Buffer	MSD	R60TX-2
Calcein AM	Thermo Fisher	C1430
Draq7	BD	564904
AMPure XP	Beckman Coulter	A63880

**Critical commercial assays**		

CellTiter-Glo®2.0	Promega	G9242
GAPDH assay kit	MSD	MSD-K151PWD-2
cIAP1 assay kit	MSD	F217V
Qiagen RNeasy Lipid Tissue Kit	Qiagen	74804
TaqMan Fast Advanced Master Mix	Applied Biosystems	4444556
Quantseq 3′mRNA-Seq Library Prep Kit-FWD	Lexogen	015
QuBit 1x dsDNA HS Assay Kit	Invitrogen	Q33231
High Sensitivity D1000 Reagents	Agilent	5067–5585
High Sensitivity DNA ScreenTape	Agilent	5067–5584
Kapa Library Quantification Kit ROX low	Roche	KK4973
Tumor Dissociation Kit, mouse	Miltenyi	130-096-730
CD45 (TIL) MicroBeads, mouse	Miltenyi	130-110-618
BD Ms Single Cell Sample Multiplexing Kit	BD	633793
BD Rhapsody Cartridge Reagent Kit	BD	633731
BD Rhapsody Cartridge Kit	BD	633733
BD Rhapsody cDNA Kit	BD	633773
BD Rhapsody Immune Response Panel Mm	BD	633753
BD Rhapsody Targeted mRNA and AbSeq Amplification Kit	BD	633774

**Deposited data**		

Single cell sequencing data GEO accession number	GSE269602	

**Experimental models: Cell lines**		

Full list of human cell lines and culture conditions is given in Table S1.		

**Experimental models: Organisms/strains**		

BomTac:NMRI-*Foxn1^nu^* mice	Taconic Biosciences	
C57BL/6NTac mice	Taconic Biosciences	
NOD SCID	Axis Bioservices	

**Software and algorithms**		

Incucyte® S3 Software	Sartorius	
Prism 10	GraphPad Software	
FlowJo	BD	
Compass for Simple Western	ProteinSimple	version 6.1
SevenBridges platform		version 1.9.1
Seurat package		version 4.2.0
Scrublet		version 0.2.3
SingleR package		version 2.0.0

### Experimental model and study participant details

#### Cell lines

If not otherwise specified, tumor cell lines were obtained from the American Type Culture Collection (Manassas, US500 VA) and were cultured in recommended media as listed in Table S1. The MKN-45 cell line was obtained from DSMZ (German Collection of Microorganisms and Cell Cultures GmbH). The Pan02 cell line was in-licensed from the NIH (NIH Ref#E−050-2016). Cell lines used in this study were authenticated by short tandem repeat (STR) analysis. All cell lines were cultivated at 37°C and 5% CO2 during the experiments.

#### Patient-derived organoids

Tumor samples used for the establishment of patient-derived organoids were obtained from patients treated at Niguarda Cancer Center, (Milano, Italy). All patients signed a dedicated informed consent in accordance with guidelines of the ALFAOMEGA Master Observational Trial (NCT04120935, IFOMCPO003/2018/PO002) or the “Analisi farmacogenomica di campioni tumorali” protocol (version 2, dated January 14th 2010). Further information on these clinical samples is provided in Table S6. These studies were conducted in accordance with the Declaration of Helsinki and under the approval of the local Independent Ethical Committee (for ALFAOMEGA study: Ethical Committee Niguarda Cancer Center Milano Area 3, decision n. 617–122018 dated December 13th 2018; for “Analisi farmacogenomica di campioni tumorali” study, decision n. 02/01_2010 dated February 10th 2010).

#### *In vivo* studies

Mice were group-housed (8–10 mice per cage) under pathogen-free and controlled environmental conditions (21 ± 1.5 C temperature, 55 ± 10% humidity) in an AAALAC accredited facility and handled according to the institutional, governmental and European Union guidelines (Austrian Animal Protection Laws, GV-SOLAS and FELASA guidelines). Animal studies were approved by the internal ethics committee and the local authorities. For establishment of CDX models, 5 × 10^6^ BxPC-3 or 1 × 10^7^ LoVo tumor cells were injected subcutaneously into 6 week-old female BomTac:NMRI-*Foxn1*^*nu*^ mice purchased from Taconic, Denmark. For establishment of the Pan02 syngeneic model, 5 × 10^6^ Pan02 cells were injected subcutaneously into female C57BL/6NTac mice purchased from Taconic Biosciences. Selected studies (C80 and LIM2551 models) were performed by Axis Bioservices according to protocols approved by the Axis Bio Animal Welfare and Ethical Review Committee, and all procedures were carried out under the guidelines of the Animal (Scientific Procedures) Act 1986. C80 and LIM2551 models were established in female NOD SCID mice by subcutaneous flank implantation of 1 × 10^7^ tumor cells (1:1 in Matrigel).

Mice were randomly distributed between the treatment and the vehicle control groups once tumors were established. Compounds were suspended in 0.5% Natrosol and administered QD intra-gastral by gavage needle. Tumor volume was determined three times a week using a caliper. The volume of each tumor [in mm^3^] was calculated according to the formula “tumor volume = length ∗ diameter2 ∗π/6.” Median of the tumor volume of each treatment group T was referred to the median of the control C as tumor growth inhibition TGI) defined as: TGI = 100 x {1-[(treated _final day_ – treated _day1_)/(control _final day_ – control _day1_)]}. Body weight was measured three times a week as an indicator of tolerability.

### Method details

#### Compounds and reagents

BI 894999 and BI 891065 are compounds developed and synthesized by Boehringer Ingelheim. TNF inhibitor Etanercept (Enbrel ) was purchased from Pfizer. Caspase inhibitor Z-VAD-FMK was purchased from Sigma-Aldrich, Cat.no. V116-2MG. Necrostatin-1 was purchased from MedChemExpress, cat.no. HY-15760.

#### Cell proliferation assay and drugs combination studies

The effect of BETi, SMACm alone or in combination cell viability was determined using a 96-h CellTiter-Glo2.0 proliferation assay. Cells were seeded in assay plates and placed at 37°C with 5% CO2 for twenty-four hours before treatment. Compounds were added in triplicate in gradients of increasing concentrations, in case of SMACm ranging between 4.5 nM and 10 μM and in case of BETi between 1.4 nM and 3 μM. DMSO served as control. Cell viability was measured by luminescence analysis using luminescence read on Envision plate readers (PerkinElmer). An additional set of colorectal cancer cell lines was assayed with technical duplicates and experimental triplicates. Cell viability was measured by luminescence analysis using luminescence read on Tecan Plate reader. The effects of drug combinations were analyzed using the Bliss Independence Model.^34^ Synergy score was calculated by the average of 3 highest and 3 lowest BLISS Gaps between predicted and measured POC (Percentage of Control) values.

For selected cell lines, *in vitro* cell growth was assessed through live-cell imaging with Incucyte S3 Live-Cell Analysis. Cells were incubated with indicated reagents in 96-well flat-bottom microtiter plates at 37°C, 5% CO2 and analyzed for 7 days by automatic imaging of 4 sectors/well in 4h intervals and analyzed using IncuCyte S3 software. Mean confluency was calculated and plotted.

#### Growth inhibition studies in patient-derived organoids

The PDOs were established and maintained in the culture as described in full details previously.^49^ Organoids were enzymatically dissociated using TrypLE Express Enzyme for 15 min at 37°C to obtain single-cell suspensions, and seeded at a density of 3,000 to 6,000 cells per well in 96-well plates precoated with basement membrane extract (BME; R&D Systems) overlayed with 100 mL of growth media containing 2% BME.The treatment with drugs started on day 3–4 after seeding when formed growing organoids were visible. Organoids were treated in fresh 150 mL of advanced DMEM/F-12 medium (Gibco) supplied with 50μg/ml primocin (InvivoGen), 2nM glutamax (Gibco), and 10nM HEPES (Euroclone) containing 2% BMEwith indicated drugs. Treatment was done automatically by Tecan D300e Digital Dispenser. A total of 4 μmol/L MG-132 was used as a positive control; DMSO served as negative control. The viability was assayed at the end of the experiment after 7 days of treatment by CellTiter-Glo (CTG) Luminescent Cell Viability assay (Promega) with modifications. Briefly, plates were equilibrated at room temperature for 30 min and reagent was mixed 1:1 with organoid media. Organoids were subjected to lysis by shaking for 25 min and then resting for 20 min. Readout was done by GloMax plate reader (Promega). The raw CTG values were normalized to the mean of the DMSO control wells. The control wells were used to calculate Z factors to indicate the quality of the data generated in the screening plate (as a standard rule, data obtaining Z factor >0.5 are acceptable). All experiments were independently repeated at least two times, and final results are expressed as an average of biological replicates.

#### Protein capillary immunoassay

Cell lysates for protein analysis were obtained by lysis of cells in RIPA buffer supplemented with 1x Halt protease and phosphatase inhibitor cocktail (Thermo Fisher Scientific, Cat.no. 78440). Cell lysates were cleared by centrifugation at 16 000 g for 20 min at 4°C. Protein concentration was determined by Bradford assay and samples were diluted to a final concentration of 0.5 mg/mL. Proteins of interest were then analyzed by using an automated quantitative capillary-based immunoassay platform, Jess and WES (ProteinSimple). Capillary immunoassays were carried out using the anti-rabbit detection module according to the manufacturer’s protocol. The following primary antibodies were used: c-IAP1 (dilution 1:50, Cell Signaling, Cat.no. 7065), c-IAP2 (dilution 1:20, Cell Signaling, Cat.no. 3130), anti-XIAP (dilution 1:50, Cell Signaling, Cat.no. 14334) anti-PARP (dilution 1:50, Cell Signaling, Cat.no. 9542), Caspase-3 (dilution 1:50, Cell Signaling, Cat.no. 14220), Cleaved Caspase-3 (dilution 1:25, Cell Signaling, Cat.no. 9664). In cases where multiplex immunoassays were performed, anti-GAPDH antibody (dilution 1:25, Abcam, Cat.no. 8245) was detected with fluorescent secondary antibody (Goat anti-Mouse IgG (H + L) Secondary Antibody [DyLight 650], Novus Cat.no. NBP1-75147C, dilution 1:50). Immunoassays were analyzed using the Compass for SW 6.1.0.

#### *Ex vivo* cIAP1 protein quantification

For biomarker analysis, BxPC-3 tumors were taken from BomTac:NMRI-Foxn1nu mice, 4 h after the treatment with 0.5% Natrosol, 2mg/kg BI 894999, 50mg/kg BI891065 or co-treatment with 2mg/kg BI 894999 + 50mg/kg BI891065. Tumors were lysed with MSD Tris Lysis Buffer (MSD, cat. R60TX-2) supplemented with protease and phosphatase inhibitors, homogenated using a tissue lyser and subsequently cleared by centrifugation. cIAP1 and GAPDH protein levels were determined using MSD Gapdh and cIAP1 assays (MSD-K151PWD-2 and F217V with 20μg Protein/well of a 96-well plate) according to the manufacturer’s instructions. Assay results were analyzed using SECTOR Imager 6000 from Meso Scale Discovery.

##### RNA purification

Total RNA was extracted from tumors using Qiagen RNeasy Lipid Tissue Kit (cat.74804, Qiagen) per manufacturer’s instructions with on-column DNase digestion. RNA concentration was measured using Quant-iT (cat.Q10213, Thermo Fisher) and RNA integrity was measured with Agilent 4200 Tapestation instrument using the Agilent RNA ScreenTape and Sample Buffer (cat.5067-5576 and 5067–5577, Agilent).

##### qRT-PCR

Synthesis of cDNA was performed on the Veriti 96 well Fast Thermal Cycler from 1 μg of total RNA using the SuperScript IV VILO Master Mix Kit (cat.11756050, Thermo Fisher) per manufacturer’s instructions. Specifically, 1 μg of total RNA was mixed with 4 μL of SuperScript IV VILO Master Mix, increasing the final reaction volume to 20 μl. The mixture was incubated for 10 min at 25°C, 10 min at 50°C, and 5 min at 85°C. The resulting cDNA diluted to 100 μl and was stored at −20°C.

The qRT-PCR assays were performed using the TaqMan Fast Advanced Master Mix (cat.4444556, Applied Biosystems) and off the shelf Taqman assays for the following genes: BIRC2, HEXIM1, TNF, GADPH, RPS18, RPL19 and HPRT1 (cat# Hs01112284, Hs00538918, Hs00174128, Hs02786624, Hs01375212, Hs02338565, Hs02800695 respectively). Each reaction contained 5 μl of 2x TaqMan Fast Advanced Master Mix, 0.5 μl of Taqman assay, 1 μl of diluted cDNA and 3.5 μl of nuclease-free water. Amplifications were carried out in 384-well plates in the QuantStudio 12K Flex PCR instrument. Thermo cycling conditions are as follows: 20 s at 95° for polymerase activation, followed by 40 cycles of 1 s denaturation at 95° and 20 s anneal/extension at 60°. Fluorescence measurements were taken at 60°C of each cycle. Analysis of qPCR data was performed using the double delta Ct method (Thermo Fisher).

##### Library generation

Libraries were prepared from the extracted RNA using Quantseq 3′mRNA-Seq Library Prep Kit-FWD (cat. 015, Lexogen) per manufacturer’s instructions using 500ng of RNA per library. Indices from columns 1–9 of the Lexogen i7 6nt Index Plate for Illumina adapters 7049–7096 were used, and 14 cycles of library amplification were performed. Library concentration was quantified using the QuBit 1x dsDNA HS Assay Kit (cat. Q33231, Invitrogen). The libraries’ average size was determined by Agilent 4200 Tapestation Instrument using the Agilent High Sensitivity D1000 ScreenTape and Sample Buffer (cat.5067-5584 and 5067–5585). The molar concentration of cDNA molecules in the individual 3′-LEXO libraries was calculated from the double stranded DNA concentration and the region average size. Equal molar of each library was combined to a pooled library. The final molarity was assessed by the Kapa Library Quantification Kit ROX low (cat KK4973, Roche Sequencing). The pooled libraries were sequenced in an Illumina NextSeq550 instrument (Illumina).

#### Preparation of single cell suspensions

15 days post treatment start, explanted Pan02 tumors were minced into small ∼1mm^3^ pieces and single cell suspensions were prepared using the Tumor Dissociation Kit, mouse (Miltenyi, 130-096-730) and the gentleMACS Octo Dissociator (Miltenyi, 130-096-427) following manufacturer’s protocol using the programs “37_m_TDK_2” and “m_impTumor_01”. Red blood cells were lysed by incubating resuspended pellets for 1–2 min on ice in 1 mL ACK lysing buffer (Thermo Fisher, A10492-01). 7 dissociated tumors per group were used for analysis by flow cytometry and 3/group were additionally enriched for tumor infiltrating leukocytes with CD45 (TIL) MicroBeads, mouse (Miltenyi, 130-110-618) using the MultiMACS X Separator (Miltenyi) followed by single cell labeling for CITE-seq analysis.

#### Flow cytometry

Approximately 1.5 × 106 total cells/tumor were stained for flow cytometry in round-bottom 96-well format. Single cell suspensions were treated with 200 U DNAse I for 10min on room temperature in 1xDPBS (Gibco, 14190-094) containing 5 mM MgCl2 (Sigma, 68475-100ML) prior to staining procedure. For exclusion of dead cells, suspensions were stained with 1:2000 diluted Fixable Blue Dead Cell Stain (Invitrogen, L34962) for 30 min on 4°C. Interactions of Fc receptors were blocked using TruStain FcX PLUS antibody (Biolegend, 156604) according to manufacturer’s recommendations. Surface marker staining was carried out by resuspending the suspensions in master mix of antibodies for extracellular antigens and incubated on 4°C for 30 min in the dark. After two washes, cells were fixed and permeabilized using the eBioscience Foxp3/Transcription Factor Staining Buffer Set (Invitrogen, 00-5523-00) according to manufacturer’s protocol. Permeabilized cells were additionally blocked with TruStain FcX PLUS antibody (Biolegend, 156604) to block any possible intracellular Fc receptor. Intracellular markers were stained by resuspending cells in master mix of antibodies specific for intracellular antigens. All reference controls and fluorescence-minus-one (FMO) controls were treated in parallel to the samples. Reference controls were generated by staining with antibodies with matching fluorophores specific for either CD45, MHCII or CD4 on TILs or beads. FMO controls were derived from TILs and contained all antibodies except the one in question and were used for gating of specific markers (Figure S4C). All antibodies were purchased with the respective fluorophore already conjugated, except Ly-6G (Biolegend, 127601) which was conjugated in-house with the NovaFluor Yellow 660 Conjugation Kit (Thermo Fisher, K06T04L007) according to manufacturer’s protocol. After washes, all stained cell suspensions were recorded using an Aurora spectral analyzer (Cytek). Unmixing of raw data was achieved with the SpectroFlo software. Data was analyzed for the markers shown in Table S4 with FlowJo (BD).

#### CITE-seq using BD rhapsody

For CITE-Seq analysis, single cell libraries were generated using the BD Rhapsody system. Enriched CD45^+^ TILs from 3 tumors/group were stained for multiplexing and with AbSeq Ab-Oligos according to manufacturer’s protocol (BD Doc ID: 214419 Rev 2.0) with slight modifications. In detail, cells were labeled with the Mouse Immune Single-Cell Multiplexing Kit, (BD, 633793), followed by equal pooling of 3 samples each (resulting in 4 pooled samples), Fc blocking with TruStain FcX PLUS antibody (Biolegend, 156604) and staining with oligo-conjugated Ab-Seq antibody mix (see Table S4 for used antibodies). Single cell capture and cDNA synthesis was performed with the BD Rhapsody Single-Cell Analysis System and the Cartridge Reagent Kit (BD, 633731), the Cartridge Kit (BD, 33733) and the cDNA Kit (BD, 633773) according to manufacturer’s protocol (BD Doc ID: 210966 Rev. 1.0). Libraries were generated with the Targeted Amplification Kit (BD, 633774) and the Mouse Immune Response Panel (BD, 633753) according to manufacturer’s protocol (Doc ID: 214508 Rev. 3.0). Pooling of libraries of each cartridge was calculated following instructions given in BDs’ “SEQ Calculator v1.2.xlsm” and libraries were sequenced (paired-end) on the Illumina platform.

Sequence alignment was performed by SevenBridges platform 1.9.1 (BD Rhapsody workflow reversion 13). The following steps were performed for the filtering of the single cell dataset: single cell with multiple or no assigned sample barcodes were discarded. Scater and scuttle packages [https://doi.org/10.1093/bioinformatics/btw777] were used to discard the cells that fail the quality control with perCellQCMetrics function. Low-quality features (both genes from scRNA-seq and CITE-seq barcodes) were detected and discarded with perFeatureQCMetrics from scuttle package. In addition, features detected in less than 10% of cells were removed. After filtering, the single cell dataset contained 30064 cells with RNA-seq readout for 266 genes and 51 CITE-seq probes for surface proteins. Single-cell datasets were analyzed with Seurat package (version 4.2.0) in R in a standard way: first 30 principal components were used for the UMAP embedding, Leiden clustering was performed with the resolution of 0.8. Scrublet (version 0.2.3) was used to detect potential doublet, cells with scrublet score smaller than 0.15 were excluded from the analysis. Automated annotation of the cell types was performed with SingleR package (version 2.0.0). With Seurat, differentially expressed genes were discovered within cell types between treatments. For visualizations, we used standard Seurat normalization of gene expression.

### Quantification and statistical analysis

#### *In vitro* studies with cell lines and patient-derived organoids

The effects of drug combinations in the cell line panel were analyzed using the Bliss Independence Model.^34^ Synergy score was calculated by the average of 3 highest and 3 lowest BLISS Gaps between predicted and measured POC (Percentage of Control) values.

For statistical analysis of *in vitro* experiments evaluating the mode of cell death in selected cell lines, one-way ANOVA with Šídák multiple comparison test was used.

Statistical analysis of experiments involving patient-derived organoids was performed using One-way ANOVA with Dunnett’s correction for multiple comparisons.

#### *In vivo* studies in mice

Statistics for the experiments evaluating the efficacy of drugs combination in mice were done using Kruskal-Wallis test with Dunn’s correction for multiple comparisons.

Statistical analysis of flow cytometry experiments was performed using one-way ANOVA with Dunnett’s multiple comparisons test.

## Supplemental information

Document S1. Figures S1–S5

Table S1. Cell lines

Table S2. IC50 synergy scores

Table S3. RNA-seq BxPC-3 *in vivo* after 4 h treatment

Table S4. Flow and AbSeq antibodies

Table S5. DEGs from CITE-seq analysis

Table S6. PDO information

Document S2. Article plus supplemental information
