# Supplementary material for: Combining BET inhibition with SMAC mimetics restricts tumor growth and triggers immune surveillance in preclinical cancer models
Source: Cell Rep Med. 2025 Aug 25;6(9):102313. doi: 10.1016/j.xcrm.2025.102313 (PMC12490239; doi:10.1016/j.xcrm.2025.102313)
Supplement: Document S1. Figures S1–S5 [file mmc1.pdf]

Cell Reports Medicine, Volume *6*

Supplemental information

Combining BET inhibition with SMAC mimetics restricts tumor growth and triggers immune surveillance in preclinical cancer models

Ksenija Slavic Obradovic, Florian Ebner, Artem V. Artemov, Martina Miotto, Paula-Elena Traexler, Robin Jacob, Ha Pham Thi Thanh, Regina Ruzicka, Andreas Wernitznig, Ines Baumann, Daniel Gerlach, Maria-Antonietta Impagnatiello, Salvatore Siena, Mary Murphy, Reniqua House, Ulrich Reiser, Valeria Santoro, Johannes Popow, Sebastian Carotta, Anke Baum, Jesse Lipp, Alberto Bardelli, Ulrike Tontsch-Grunt, Mariangela Russo, and Martin Aichinger

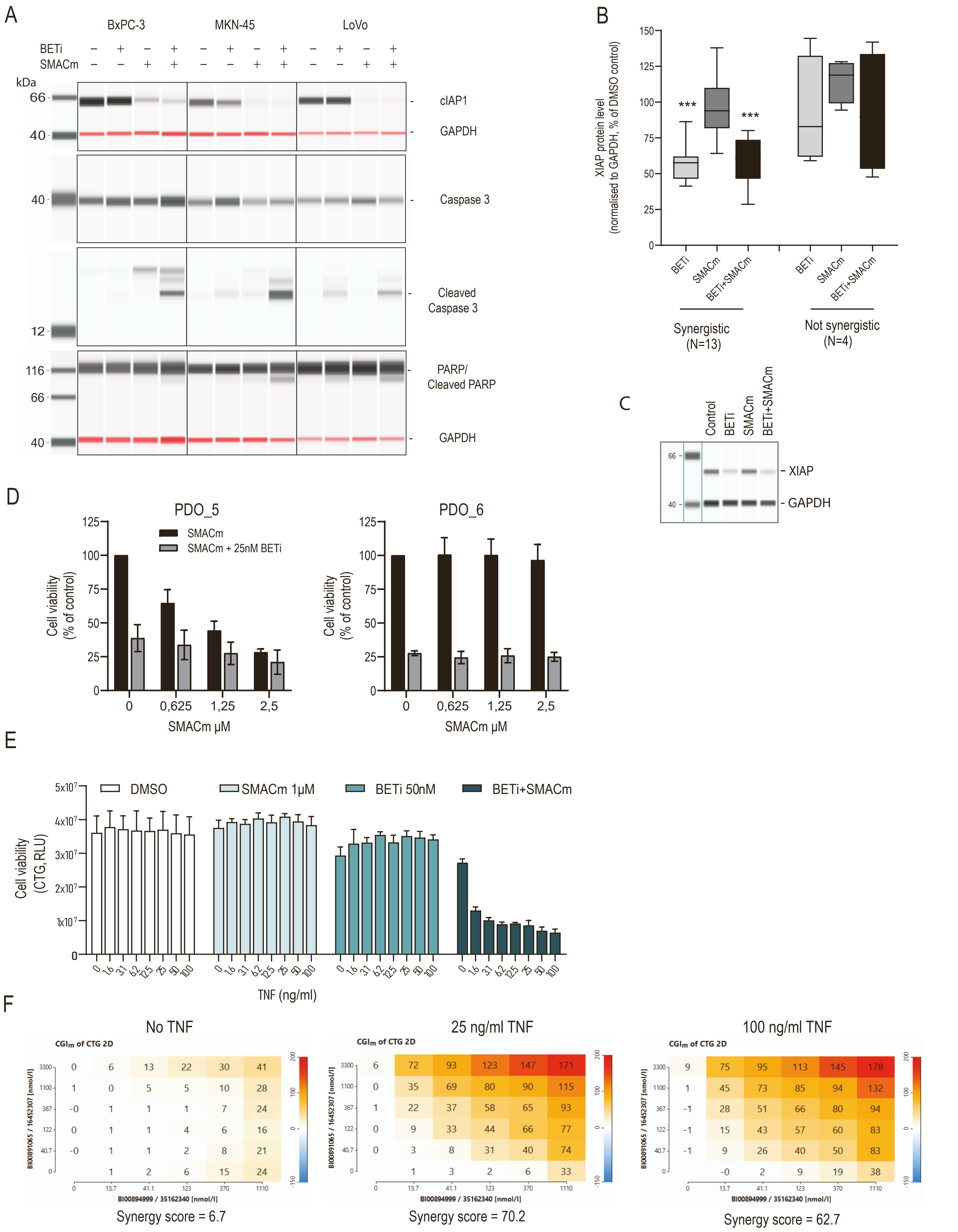

Supplementary figure 1. ***In vitro*** **characterization of SMACm and BETi effects on cancer cell proliferation and cell death induction**

A. JESS (Simple Western System) analysis of Caspase 3 and PARP cleavage and cIAP1 levels in BxPC-3, MKN-45 and LoVo cell lines upon treatments with BETi and SMACm at indicated concentrations. Cell pellets were collected for lysis and JESS analysis at 24 h timepoint after treatments start. **B.** BETi down-modulates XIAP protein expression across a panel of cell lines (300nM BETi and 1000nM SMACm for 24h), grouped into synergistic (N=13) and non-synergistic (N=4) cell lines. Statistics: one sample Wilcoxon test (two-tailed) **C.** Example of WES (Simple Western System) analysis of XIAP and GAPDH levels upon treatment of BxPC-3 cells with 300nM BETi and 1000nM SMACm for 24h. **D.** Effect of combinatorial treatment on two CRC patient-derived organoids; shown are mean ± SEM values from 3 independent experiments, each normalized to DMSO control (additional PDO models to those shown in the Fig.3) **E.** *In vitro* Pan02 sensitivity to TNF alone (concentrations indicated on the graph) or in the presence of BETi (50nM), SMACm (1µM) or BETi+SMACm combination. Cell viability was measured by CTG assay 96h upon treatments start. **F.** Proliferation assay for synergy analysis in Pan02 cell line in the absence or presence of 25ng/ml or 100ng/ml mouse TNF; shown is combination plate map matrix and the cell growth inhibition (CGI) at the indicated concentrations. CGI values <100%: tumor cell growth, CGI 100%: tumor cell stasis and GCI > 100%: tumor cell killing. Shown are Bliss Gap analysis synergy scores calculated for the BETi+SMACm combination.

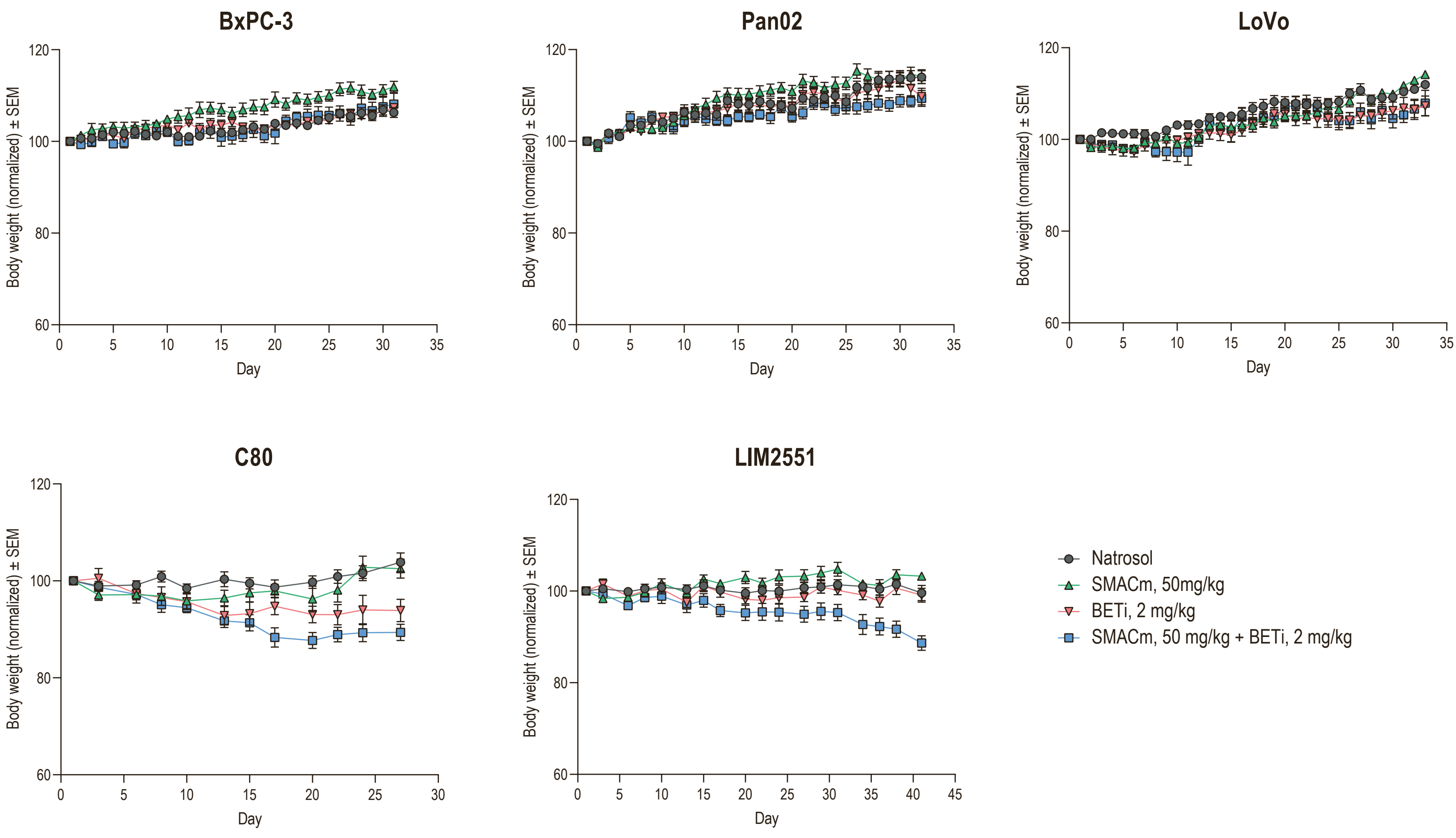

Supplementary figure 2. Normalized body weight measurements for *in vivo* xenograft models shown in Figure 3.

A

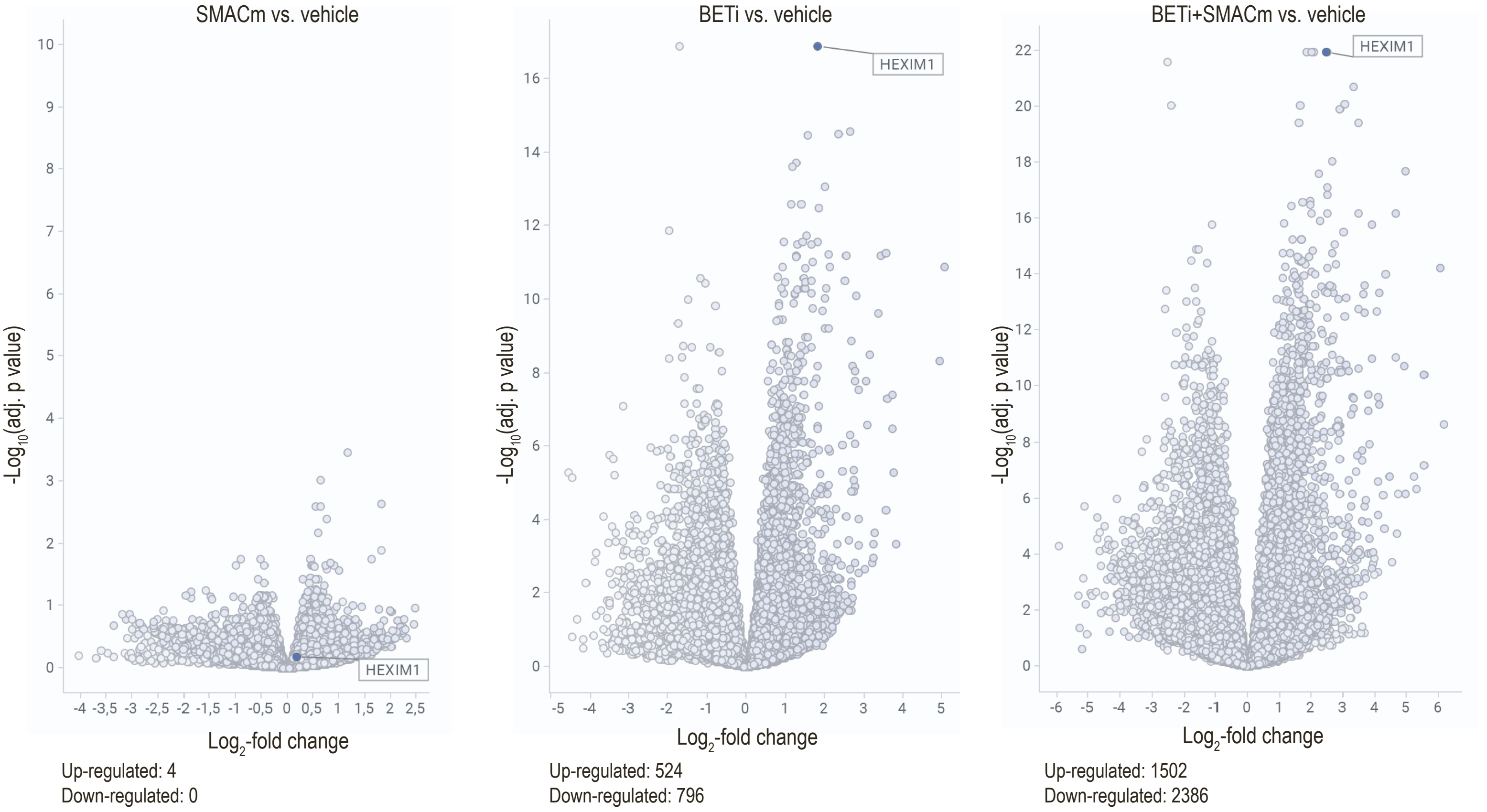

B

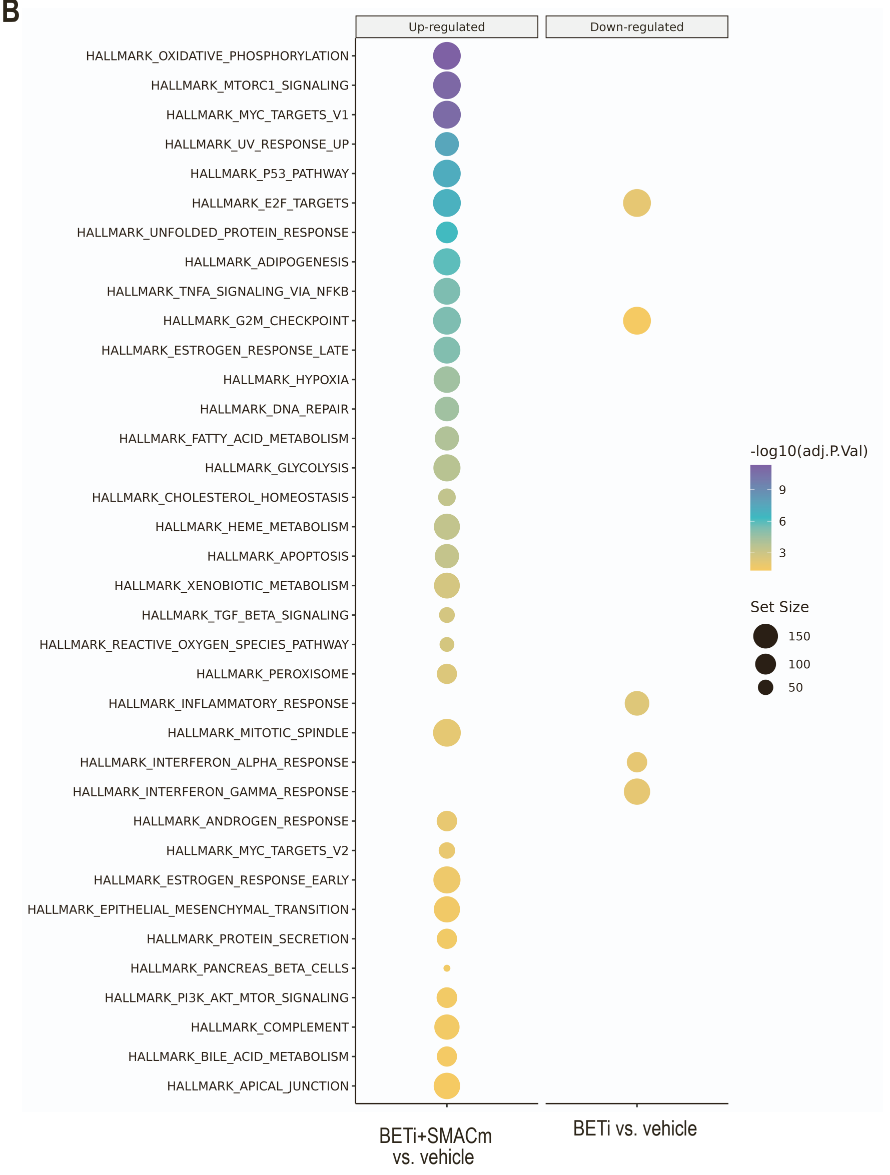

C

GSEA of BETi+SMACm vs. BETi DEGs

Term	Set_size	Direction	P.value	Adj.p.value	Significant
HALLMARK_E2F_TARGETS	199	greater	5,85E-15	2,92E-13	Up-regulated
HALLMARK_MYC_TARGETS_V1	199	greater	1,74E-14	4,36E-13	Up-regulated
HALLMARK_OXIDATIVE_PHOSPHORYLATION	197	greater	3,15E-13	5,25E-12	Up-regulated
HALLMARK_MTORC1_SIGNALING	197	greater	3,52E-12	4,4E-11	Up-regulated
HALLMARK_G2M_CHECKPOINT	199	greater	5,91E-11	5,91E-10	Up-regulated
HALLMARK_TNFA_SIGNALING_VIA_NFKB	182	greater	5,73E-10	4,78E-09	Up-regulated
HALLMARK_P53_PATHWAY	195	greater	1,9E-07	0,00000136	Up-regulated
HALLMARK_ESTROGEN_RESPONSE_LATE	183	greater	7,91E-07	0,00000494	Up-regulated
HALLMARK_ADIPOGENESIS	187	greater	1,14E-06	0,00000635	Up-regulated
HALLMARK_UV_RESPONSE_UP	140	greater	5,17E-06	0,0000258	Up-regulated
HALLMARK_FATTY_ACID_METABOLISM	143	greater	2,59E-05	0,000118	Up-regulated
HALLMARK_HYPOXIA	178	greater	6,37E-05	0,000265	Up-regulated
HALLMARK_APOPTOSIS	144	greater	7,81E-05	0,000297	Up-regulated
HALLMARK_UNFOLDED_PROTEIN_RESPONSE	110	greater	8,31E-05	0,000297	Up-regulated
HALLMARK_DNA_REPAIR	148	greater	0,000159	0,000532	Up-regulated
HALLMARK_EPITHELIAL_MESENCHYMAL_TRAN	172	greater	0,00042	0,00131	Up-regulated
HALLMARK_INTERFERON_GAMMA_RESPONSE	174	greater	0,000475	0,0014	Up-regulated
HALLMARK_MYC_TARGETS_V2	58	greater	0,00051	0,00142	Up-regulated
HALLMARK_CHOLESTEROL_HOMEOSTASIS	68	greater	0,000714	0,00188	Up-regulated
HALLMARK_XENOBIOTIC_METABOLISM	167	greater	0,000993	0,00248	Up-regulated
HALLMARK_MITOTIC_SPINDLE	198	greater	0,00128	0,00305	Up-regulated
HALLMARK_REACTIVE_OXYGEN_SPECIES_PATH	46	greater	0,00258	0,00566	Up-regulated
HALLMARK_INFLAMMATORY_RESPONSE	149	greater	0,0026	0,00566	Up-regulated
HALLMARK_APICAL_JUNCTION	176	greater	0,00272	0,00566	Up-regulated
HALLMARK_HEME_METABOLISM	170	greater	0,00288	0,00576	Up-regulated
HALLMARK_GLYCOLYSIS	187	greater	0,00401	0,00758	Up-regulated
HALLMARK_INTERFERON_ALPHA_RESPONSE	95	greater	0,00409	0,00758	Up-regulated
HALLMARK_KRAS_SIGNALING_DN	128	anydir	0,000187	0,00937	Any direction
HALLMARK_COMPLEMENT	158	greater	0,00839	0,015	Up-regulated
HALLMARK_PANCREAS_BETA_CELLS	21	greater	0,00949	0,016	Up-regulated
HALLMARK_ALLOGRAFT_REJECTION	131	greater	0,00959	0,016	Up-regulated
HALLMARK_ESTROGEN_RESPONSE_EARLY	187	greater	0,0163	0,0262	Up-regulated
HALLMARK_TGF_BETA_SIGNALING	53	greater	0,0183	0,0283	Up-regulated
HALLMARK_ANDROGEN_RESPONSE	96	greater	0,0187	0,0283	Up-regulated
HALLMARK_COAGULATION	97	greater	0,0212	0,0312	Up-regulated
HALLMARK_PEROXISOME	93	greater	0,0257	0,0368	Up-regulated
HALLMARK_IL2_STAT5_SIGNALING	170	greater	0,0297	0,0412	Up-regulated

Supplementary figure 3. RNAseq analysis of BxPC-3 *in vivo* xenograft model

A. RNAseq analysis of BxPC-3 tumors obtained from *in vivo* biomarker experiment, 4h time-point. Experiment included 5 natrosol-control replicates and 3 replicates for all other groups. **B**. Comparison of Gene set enrichment analyses (GSEA) of differentially expressed genes upon BETi+SMACm and BETi treatments shown in A. **C**. Table showing GSEA of differentially expressed genes in BETi+SMACm vs. BETi analysis.

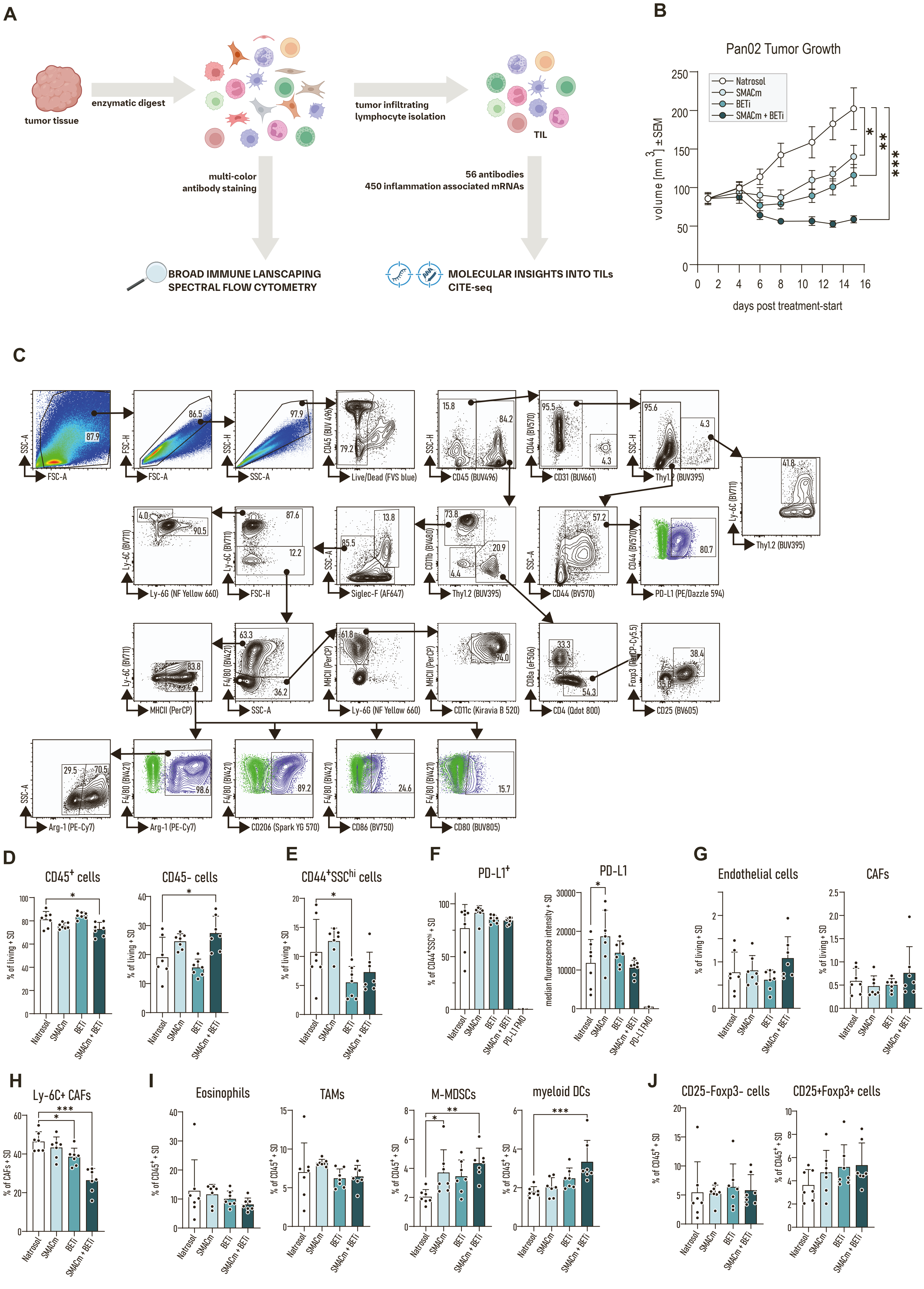

Supplementary figure 4. Flow cytometry analysis of Pan02 tumors obtained from *in vivo* Pan02 syngeneic model

A. A scheme representing the experimental design of the Pan02 *in vivo* experiment for tumor microenvironment analysis by flow cytometry and CITE-seq readouts. **B.** Tumor growth curves from experiment shown in A (n=10/group). Mice were injected subcutaneously with 5x106 Pan02 cells and daily gavaged with Natrosol, SMACm 50mg/kg, BETi 2mg/kg or SMACm 50mg/kg + BETi 2mg/kg. **C.** Gating strategy applied to define depic-ted cell types (see shaded gates and names). **D.** Bar plots depicting immune (CD45+, left) and non-immune (CD45-, right) cells in the tumor microenvironment. E. Frequency of CD45-CD31-Thy1.2-CD44+SSChi cells among all living cells. **F.** CD44+SSChi cells expressing PD-L1. Left shows fraction of positive cells and right shows median fluorescence intensity. **G.** Frequency of endothelial cells and cancer associated fibroblasts (CAFs) of living cells gated as living CD45-CD31+ and living CD45-CD31-Thy1.2+, respectively. **H.** Fraction of CAFs expressing Ly-6C. **I.** Myeloid cell populations as fraction of all immune cells. Eosinophils were gated as living CD45+CD11b+Siglec-F+SSChi, TAMs as living CD45+CD11b+-Siglec-F-Ly-6C-F4/80+MHCII+, monocytic myeloid derived suppressor cells (M-MDSCs) as living CD45+CD11b+Siglec-F-Ly-6ChiLy-6G- and myeloid dendritic cells (myeloid DCs) as living CD45+CD11b+Siglec-F-Ly-6C-F4/80-MHCII+CD11c+. **J.** T Helper cells (CD25-Foxp3-) and regula-tory T cells (CD25+Foxp3+) pre-gated on living CD45+Thy1.2+CD4+. n=7/group for flow analysis, shown is SD (one-sided) on bar plots. Statistical significance: *=p<0.05; **=p<0.01; ***=p<0.001; no label = not significant.

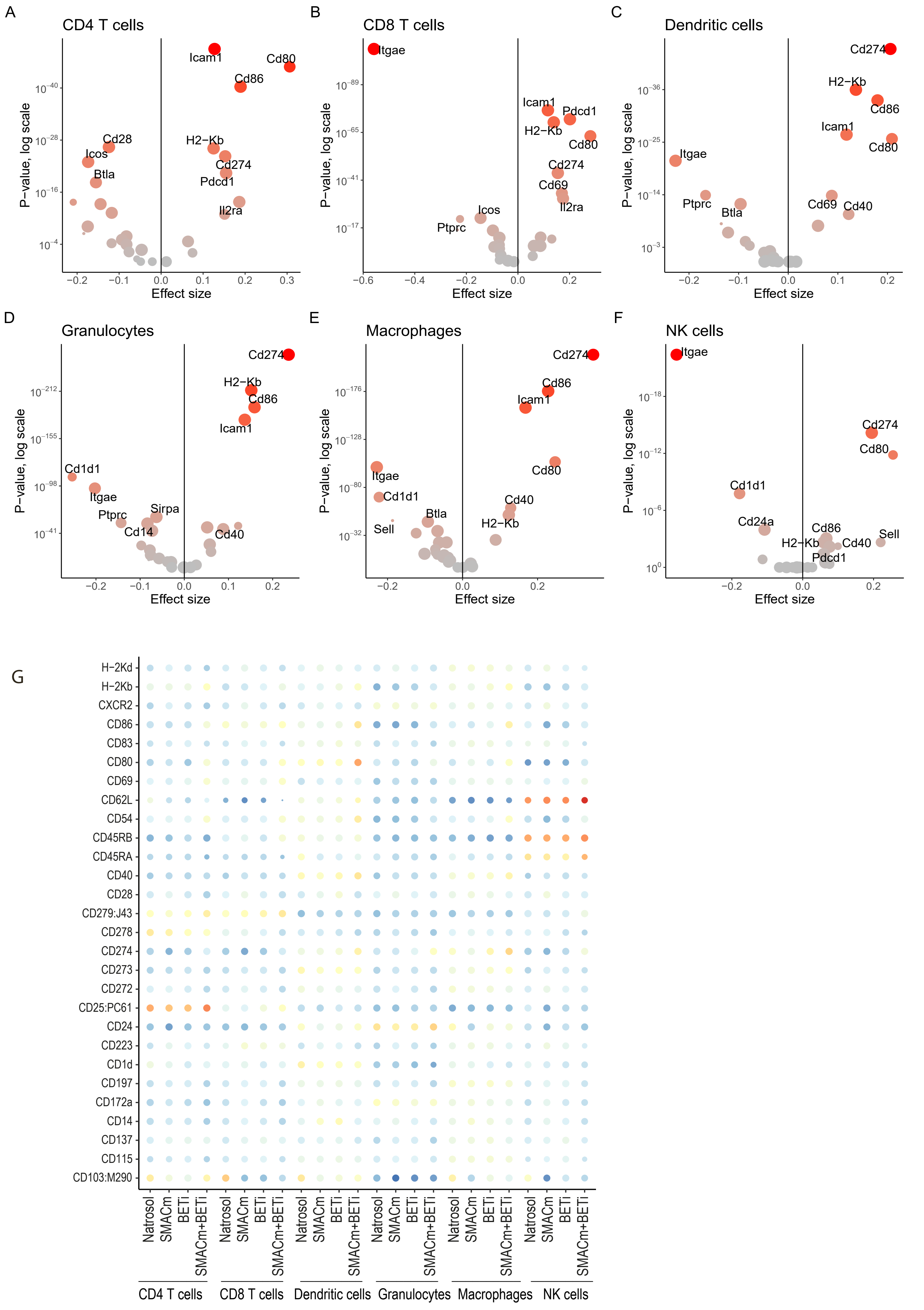

Supplementary figure 5. Protein-level CITE-seq analysis of SMACm and BETi treatment effect on the immune compartment in syngeneic Pan02 model A-F. Volcano plots showing differentially abundant surface proteins between tumors treated with BETi BI 894999 and SMACm BI 891065 vs Natrosol-treated controls in each cell type: (A) CD4+ T cells, (B) CD8+ T cells, (C) Dendritic cells, (D) Granulocytes, (E) Macrophages, (F) NK cells. X and Y axes represent log2 fold change and P-value (logarithmic scale), respectively. The color of the dots corresponds to the P-value (Y axis). **G.** Heatmap of protein markers (excluding the lineage markers) analysed in CITE-seq.
